# Fragment Merging Using a Graph Database Samples Different Catalogue Space than Similarity Search

**DOI:** 10.1021/acs.jcim.3c00276

**Published:** 2023-05-25

**Authors:** Stephanie Wills, Ruben Sanchez-Garcia, Tim Dudgeon, Stephen D. Roughley, Andy Merritt, Roderick E. Hubbard, James Davidson, Frank von Delft, Charlotte M. Deane

**Affiliations:** † Department of Statistics, University of Oxford, Oxford OX1 3LB, United Kingdom; ‡ Centre for Medicines Discovery, University of Oxford, Oxford OX3 7DQ, United Kingdom; § Informatics Matters, Ltd., Perch Coworking, Franklins House, Bicester OX26 6JU, United Kingdom; ∥ Vernalis (R&D) Limited, Granta Park, Great Abington, Cambridge CB21 6GB, United Kingdom; ⊥ LifeArc, Lynton House, 7−12 Tavistock Square, London WC1H 9LT, United Kingdom; # Diamond Light Source, Didcot OX11 0DE, United Kingdom; ∇ Research Complex at Harwell, Harwell Science and Innovation Campus, Didcot OX11 0FA, United Kingdom; ○ Department of Biochemistry, University of Johannesburg, Auckland Park, Johannesburg 2006, South Africa

## Abstract

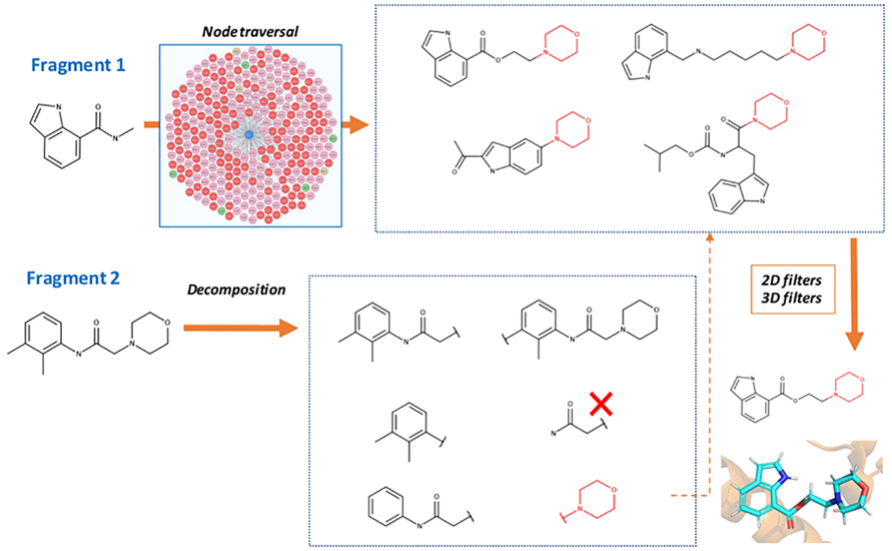

Fragment merging is a promising approach to progressing fragments directly to on-scale potency: each designed compound incorporates the structural motifs of overlapping fragments in a way that ensures compounds recapitulate multiple high-quality interactions. Searching commercial catalogues provides one useful way to quickly and cheaply identify such merges and circumvents the challenge of synthetic accessibility, provided they can be readily identified. Here, we demonstrate that the Fragment Network, a graph database that provides a novel way to explore the chemical space surrounding fragment hits, is well-suited to this challenge. We use an iteration of the database containing >120 million catalogue compounds to find fragment merges for four crystallographic screening campaigns and contrast the results with a traditional fingerprint-based similarity search. The two approaches identify complementary sets of merges that recapitulate the observed fragment–protein interactions but lie in different regions of chemical space. We further show our methodology is an effective route to achieving on-scale potency by retrospective analyses for two different targets; in analyses of public COVID Moonshot and *Mycobacterium tuberculosis* EthR inhibitors, potential inhibitors with micromolar IC_50_ values were identified. This work demonstrates the use of the Fragment Network to increase the yield of fragment merges beyond that of a classical catalogue search.

## Introduction

Fragment-based drug discovery (FBDD) involves the screening of low-molecular-weight compounds (typically ∼200 Da) against a target of interest, which can subsequently be developed into more potent binders.^1,2^ While fragments tend to bind with weak affinity (typically in the high micromolar to low millimolar range),^1^ their small size and lower likelihood of steric impediments result in increased likelihood of binding and higher hit rates compared with more traditional high-throughput screens (HTS).^3,4^ Other benefits of FBDD include smaller library sizes, the ability to cover a greater proportion of chemical space, and greater control over the optimization of desired chemical properties.^5−8^

Fragment screening using X-ray crystallography is a gold-standard technique as it directly reveals the binding mode of the fragment, providing both confirmation of binding and rich structural data to guide the progression of fragments to become potent and specific binders.^9,10^ The throughput of the technique has dramatically improved owing to advances in synchrotrons, automation, and algorithms.^11^ Nevertheless, a major bottleneck remains in how to efficiently exploit the information contained in crystal complexes for the rapid structure-guided progression of fragments. There are few efficient, fully automated workflows for this purpose,^12−14^ though they clearly hold great potential. For instance, Müller et al.^13^ used four crystallographic complexes against protein kinase A as seed fragments for the template-based docking of Enamine REAL compounds and synthesized 93 molecules, the most promising of which demonstrated an inhibition constant (K_i_) of 744 nM.

There are three main strategies for fragment progression: growing, which involves the addition of atoms or functional groups that reach new and favorable interactions; linking, which involves the design of a molecular linker to join two fragments that bind to distinct regions of the pocket; and merging, which is used for fragments that bind in partially overlapping space by designing compounds that incorporate substructural features from each.^1^ We refer to compounds that maintain exact substructures as “pure merges”.

Fragment merging remains poorly served by *in silico* approaches, and the published successful fragment merges have been manually designed.^15−22^ Not only is this not transferable, but it does not allow the full exploitation of even modest numbers of hits, relying instead on the experience and biases of the medicinal chemist. Only *in silico* approaches will be able to fully sample the relevant chemical space, though the main challenge lies in recapitulating and maintaining the orientation and interactions of the parent fragments.^23^

In contrast, molecular hybridization, which is conceptually similar to fragment merging but is used in lead optimization, has many computational tools available. Typically, these methods rely on an overlapping substructure to exist between a set of input ligands, both in terms of its chemistry and spatial organization.^24−26^ BREED^24^ was an early implementation that performs hybridization by overlaying ligands from several protein–ligand structures, identifying matching bonds between the set of ligands and swapping fragments around these matched bonds to generate new hybrid structures. Other hybridization tools use an iterative approach to molecular generation.^27−29^ Fragment shuffling^29^ aligns several protein–ligand complexes and assigns scores to ligand atoms denoting their contribution to overall binding, after which ligands are fragmented and a selected seed fragment is iteratively grown depending on the calculated fragment scores.

Looking further afield, “impure merges” do not incorporate exact substructures of the parent fragments, but instead incorporate those with similar chemotypes or pharmacophoric properties. This is related to scaffold hopping techniques, which involve the substitution of a molecular core with that of a similar chemotype. For example, Recore^30^ replaces fragments in a molecule according to their pharmacophore and arrangement of exit vectors, while CReM^31^ is a tool that can mutate, grow or link compounds by selecting fragments to be added that share the correct chemical context.

*De novo* design using generative models has become increasingly common in the field of molecular optimization, particularly for fragment growing^32−37^ and linking.^38−41^ However, there are no such existing models for performing fragment merging, partly owing to the lack of data and difficulty in generating synthetic data for training. The main limitation of all *de novo* approaches is that they tend to propose molecules with poor synthetic accessibility and thus are either impossible or difficult and expensive to make; even state-of-the-art approaches for fragment elaboration struggle to design accessible molecules.

There is a further challenge in progressing fragments into an efficient drug discovery campaign, which is that greater focus needs to be placed on finding readily available compounds to allow elucidation of the structure–activity relationship (SAR) for a target. Commercial catalogues are generally used for this purpose as the compounds are guaranteed to be synthetically feasible and are cheap and easy to acquire. Nevertheless, there are limited examples within the literature of formalized workflows that use catalogue searches to identify and filter compounds.^12,42^ Existing methods typically employ search techniques such as substructure and similarity searching,^12^ yet these methods can be computationally intensive and fingerprint-based similarity metrics can exhibit bias against fragments (as smaller molecules occupy fewer bits).

In this work, we demonstrate the use of a graph database as a method for finding fragment merges in commercial catalogues, specifically, using an iteration of the Fragment Network. The concept of the Fragment Network was first described by Astex Pharmaceuticals.^43^ In that paper, the authors mapped chemical space into a graph database and demonstrated its use for optimizing initial hits against protein kinase B and hepatitis C virus protease–helicase.^43^ In this study, we reimplemented the Fragment Network using the RDKit cheminformatics toolbox,^44^ loaded it with 120 million commercial catalogue compounds, and developed a pipeline to search for fragment merges, followed by several 2D and 3D filters that prioritize candidates by the likelihood of maintaining the binding pose and interactions of the parent fragments. Unlike other hybridization methods, this approach does not require matching overlapping substructures between an existing set of ligands.

Our Fragment Network searches yield complementary results to classical similarity searches using molecular fingerprints, as each technique identifies filtered compounds for pairs of fragments for which the other technique fails, suggesting that the two techniques should be used in parallel. Specifically, the Fragment Network naturally lends itself both to identifying pure merges and to limiting searches to local chemical space, leading to greater search efficiency. It is thus well-suited to fully exploiting all crystallographic data through large-scale enumeration of all possible pairs of fragment hits. Its suitability for fragment progression was confirmed in two retrospective analyses: comparison against experimental data from the COVID Moonshot^10^ identified a known inhibitor and close analogues to other known inhibitors with half-maximal inhibitory concentration (IC_50_) values within the low-to-medium micromolar range. A known binder and close analogues to known inhibitors were also identified using experimental data against *Mycobacterium tuberculosis* transcriptional repressor protein EthR.

## Materials and Methods

### Fragment Network Database

The version of the Fragment Network used for this work was compiled in March 2022 and contains XChem fragment libraries and compounds from the Enamine, MolPort, and Chemspace commercial catalogues, totalling >120 million compounds. The code to generate the database is publicly available at https://github.com/InformaticsMatters/fragmentor.

The network was implemented as described in the original paper:^43^ generation of the network involves the decomposition of molecules—represented as nodes—into rings, linkers, and substituents, with the iterative removal of these groups resulting in the enumeration of connected nodes. The network is populated with purchasable compounds that are connected via edges, which represent transformations between nodes. A schematic demonstrating the types of transformation that can be made is shown in Figure S1. The corresponding metadata for nodes and edges, describing features such as the substructure being removed or added when traversing an edge, or, optionally, molecular properties of the molecule node, can allow the tailoring of search queries. The XChem implementation uses Neo4j^45^ as the graph database platform, and queries are written in Cypher. This language can enable the creation of sophisticated search queries whereby a user can specify parameters such as the number of hops involved in the query (the number of hops refers to the path distance between nodes in the network), the type of hop to be used in the query path (that is, a contraction or expansion), the substructure(s) involved in the transformation, and the filtering of results based on node properties (for example, heavy atom count).

### XChem Data Sets

The four test cases are publicly available crystallographic fragment screens from the XChem facility, summarized in Table 1 and Table S1. These data are available to download from the Fragalysis platform (https://fragalysis.diamond.ac.uk/viewer/react/landing). The specific experiments are described below.

**Table 1:** Summary of Fragment Screens Used as Test Data

Target	Type of binding site	Number of fragments	Number of pairs for merging^a^
DPP11	Allosteric	11	55
PARP14	Active	13	75
nsp13	Active	9	35
Mpro	Active	19	134

a Value reflects the number of pairs after removing fragment pairs that are highly similar.

A helicase protein, SARS-CoV-2 nonstructural protein 13 (nsp13), forms part of the replication and transcription machinery of SARS-CoV-2, together with 15 other nonstructural proteins. The function of nsp13 is to catalyze the unwinding of genetic material using energy from nucleotide triphosphate hydrolysis. Two sites were found to have good ligandability in a fragment screen; for the purposes of this work, we focus on developing merges of fragments that bind to the nucleotide site, which is positioned between the 1A and 2A helicase subdomains.^46^ Nine overlapping fragment hits that offer good merging opportunities were chosen for testing the pipeline.

The SARS-CoV-2 main protease (Mpro) is involved in processing polyproteins pp1a and pp1ab of SARS-CoV-2, which are necessary for replication and transcription. Mpro is a homodimer of two polypeptides: protomers A and B. Each protomer consists of three domains, and the substrate-binding site, which consists of multiple subsites, is positioned in a cleft between domains I and II, which comprise antiparallel β-barrel structures.^47^ Twenty-three noncovalent fragments were found to bind to the active site during crystallographic screening.^48^ In this work, we focus on designing merges for 19 of these fragments; the remaining four fragments were used for an independent case study described below.

Human poly(ADP-ribose) polymerase 14 (PARP14) is part of a family of proteins involved in post-translational modification. Shared between the proteins is a highly conserved catalytic domain that binds to NAD+ and transfers negatively charged ADP-ribose to the target protein to be modified.^49^ This study focuses on 13 fragment hits found to bind to the catalytic site.

*Porphyromonas ginigivalis* plays a causative role in the development of periodontal disease.^50^ The dipeptidyl peptidase (DPP) enzymes are central to the energy metabolism of the bacterium.^50^ Fragment screening found hits that bound to two sites: the active site, to which two fragments bound, and to a potential allosteric site. While the mode of action has yet to be established, 11 fragments were found to bind to the allosteric site; the opportunity for inhibitor development means these 11 hits were selected for merging.

### Molecular Fingerprint Calculation

All molecular fingerprints were calculated using the RDKit implementation^44^ of the Morgan fingerprint (using 2048 bits and a radius of 2). This fingerprint type is used in all similarity and clustering calculations.

### Computational Workflow: Querying

**Figure 1:**
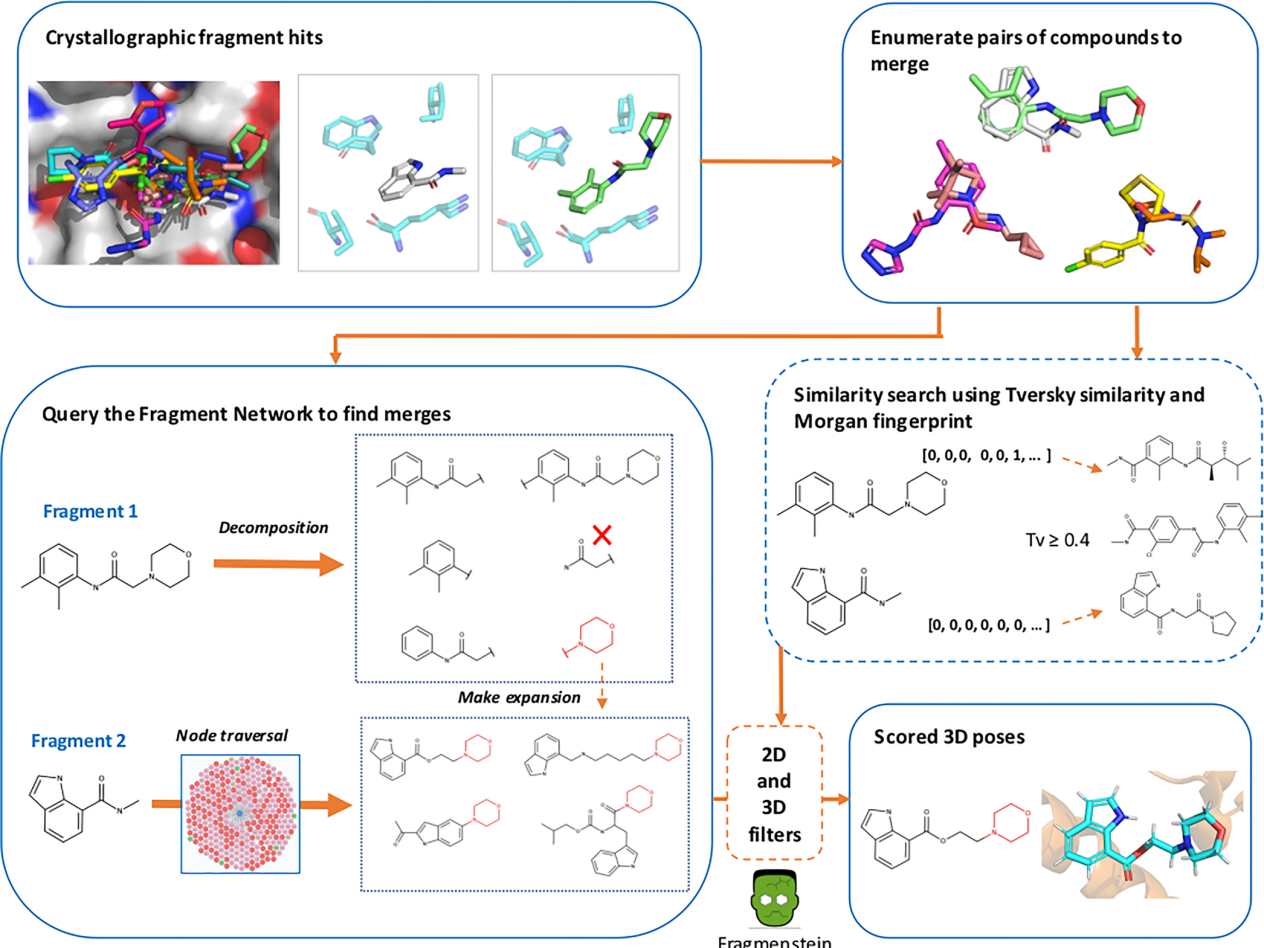
Pipeline for identifying fragment merges. Fragment hits from crystallographic fragment screens are used for finding fragment merges. All possible pairs of compounds are enumerated for merging (removing those with high similarity). Both the Fragment Network and similarity search are used to identify fragment merges. The Fragment Network enumerates all possible substructures of one of the fragments in the merge while the other fragment is regarded as the seed fragment. A series of optional hops are made away from the seed fragment (up to a maximum of two), after which an expansion is made by incorporating a substructure from the other fragment. The similarity search finds merges by calculating the Tversky (Tv) similarity against every compound in the database using the Morgan fingerprint (2048 bits and radius 2). The Tversky calculation uses α and β values of 0.7 and 0.3, respectively. All compounds with a mean similarity ≥0.4 are retained. The merges pass through a series of 2D and 3D filters, including pose generation with Fragmenstein, to result in scored poses.

The overall pipeline for querying the database and filtering compounds is illustrated in Figure 1. For all compatible pairs of fragments, we query the Fragment Network as described in the following section. We define two fragments as compatible if they are close in space and are not highly similar to each other. Only pairs for which the distance between the closest pair of atoms is <5 Å are considered. The chosen limit allows the identification of both merges and linkers; this distance threshold can be modified according to which type of elaborated compound to favor. We remove fragments that represent close analogues that bind in an equivalent position to ensure that hybrid molecules are substantially different from the parents. To do so, the maximum common substructure (MCS) between the two fragments is calculated. If up to three heavy atoms in either fragment are not included in the MCS, the RMSD is calculated between the MCS atoms, and fragment pairs are removed, by default, if the RMSD is <2 Å (these parameters can be modified by the user). These pairs typically do not represent useful merges as the resulting compounds do not incorporate unique substructures from each fragment. The total numbers of pairs to undergo querying are shown in Table 1.

#### Search by Fragment Network Query

The Fragment Network query aims to identify close neighbors of one fragment, the seed fragment, that incorporate a substructure of the other fragment in the pair. First, all possible substructures of one of the fragments in the pair are enumerated (this is done by traversing down all edges away from the fragment node that denote contractions and extracting the substructures that are removed). Substructures are filtered for those that contain at least three carbon atoms and do not exist in the other fragment in an overlapping position (considered as >50% overlap in volume). This ensures that the substructure makes a substantial and unique contribution to the final merge. Queries identify all nodes within a specified number of hops away from the seed fragment; the query then attempts to make an additional expansion hop from these nodes in which one of the substructures from the other fragment is incorporated (thus resulting in compounds that contain substructures of both original fragments).

To avoid redundancy in queries, for a single fragment, the substructures for all possible paired fragments are enumerated and pooled together (as the same substructure could be present in multiple fragments). The Fragment Network querying process is asymmetric (that is, there are two sets of queries for each pair of fragments, with each fragment undergoing expansion). The number of optional hops is a tunable parameter; increasing the number of hops will result in a deeper search but will lead to an exponential increase in the time required. In this work, we limit the number of hops to a maximum of two.

All retrieved molecules are filtered for those that contain at least 15 heavy atoms to ensure the compounds represent true merges between the fragments. Query paths (that is, the path taken between the seed fragment node and the merge compounds) are limited to those where the node before expansion contains at least six carbon atoms and are not equivalent to the substructure used in the expansion (this prevents the query from retrieving all nodes connected to the substructure or making expansions from small, ubiquitous nodes that make vast numbers of connections). A limit of 3000 molecules per fragment pair was imposed before filtering to yield numbers comparable to that of the similarity search.

As a consequence of limiting the substructure for expansions to molecules containing at least three carbons, this can result in the Fragment Network not being able to incorporate substituents from the original fragment into the final merge. To combat this, we provide an option to perform R-group expansions of the final nodes. This can be performed as part of the initial query or as a postrefinement step of the most promising filtered molecules.

#### Similarity Search

The similarity search was performed on the equivalent set of purchasable molecules available in the Fragment Network. For each pair of fragments, the Tversky similarity was calculated (using α and β values of 0.7 and 0.3, respectively) against every compound in the database. The Tversky index is an asymmetric metric and thus prioritizes whether the merges replicate the same bits as the fragments. The geometric mean was calculated between the two values, and a mean value of 0.4 was used as a cutoff (the geometric mean was used to penalize compounds that are highly similar to only one of the fragments in the pair). The number of merges per pair was limited to the 3000 with the highest similarity values (Supplementary Figure S2). Merges with <15 heavy atoms were removed to allow fair comparison with merges identified using the Fragment Network. While an alternative approach would be to perform a single query using the union of the fingerprints for the two fragments, we opted against using this approach, as this will result in smaller fragments contributing less weight to the similarity calculation.

### Computational Workflow: Filtering

The retrieved merges are passed through a set of 2D and 3D filters to retain the most promising compounds that are most likely to maintain the binding pose and interactions of the parent fragments. The same filters are applied to the merges from both the Fragment Network and similarity searches to allow fair comparison, with the exception of the expansion filter described below, which is only applied to Fragment Network-derived compounds. These filters are designed for large-scale fragment screens and are thus intentionally stringent. The pipeline has been implemented to enable flexibility: in cases where the filtering pipeline may result in few filtered compounds, it is straightforward to modify the filtering pipeline by either removing filtering steps or modifying thresholds, so that the pipeline can be used in all types of situations.

#### Filtering Using Calculated Molecular Descriptors

Compounds are filtered according to calculated molecular descriptors using Lipinski’s rule of five^51^ and a maximum rotatable bond count of ten.^52^

Furthermore, a limit on the number of consecutive nonring bonds was imposed to remove “stringy”, flexible molecules. Thresholds of eight bonds for “linkers” (substructures connecting two rings) and six bonds for “side chains” (substructures connected to only one ring) were imposed. This was done by calculating the maximum path length of the nonring substructures. Thresholds were set by analyzing the number of consecutive nonring bonds in ChEMBL29^53^ drug molecules (after applying the Lipinski and rotatable bond filters) and calculating the 95th percentile (Figure S3).

#### Filtering Compounds That Resemble Expansions of One Fragment

A filter was implemented to remove compounds that resemble “expansions” (that is, compounds that represent expansions of one fragment rather than true merges). This occurs when the fragment contributing the substructure for expansion does not contribute anything unique to the final merge. First, the MCS between the seed fragment and the merge is calculated and removed from the merge. The MCS is then calculated between the remainder of the molecule and the substructure used for expansion. If the MCS comprises at least three heavy atoms, the merge passes the filter. This filter is only applied to compounds derived from the Fragment Network approach as the search is found to result in a high proportion of these compounds when the substructure used for expansion is already present in the seed fragment.

#### Filtering Compounds Using Constrained Embedding

This filter assesses whether it is possible to generate physically reasonable conformations using distance-based constraints based on the poses of the parent fragments. The filter retrieves the atomic coordinates of substructures that were derived from the parent fragments by calculating the MCS or using the substructure that was used for expansion in the original query. The two sets of coordinates from both fragments are combined and used as a template for embedding the merge. As there may be multiple possible sets of atomic coordinates (for example, if there are several substructure matches between the MCS and the merge), every possible pair of coordinates is tried with every possible match with the merge. Merges for which it is possible to generate a physically reasonable structure using the RDKit constrained embedding implementation^44^ (that is, the bond distances fulfill the limits set by the distance bonds matrix) undergo an energy calculation using the conformation generated by the constrained embedding; the energy is calculated using RDKit^44^ using the UFF molecular force field (this force field is used for consistency with the RDKit constrained embedding implementation).

The energy of the constrained conformation is compared with the mean energy of 50 unconstrained conformations to rule out molecules with energetically infeasible poses. The conformations are randomly generated using RDKit and optimized using the UFF force field; the energy is calculated in the same way as the constrained conformations. A total of 50 conformations was deemed to be sufficient according to the rotatable bond count of the unfiltered molecules (the unfiltered data sets show an average number of rotatable bonds of less than 7).^54^ If the ratio between the two energy calculations is >7, the molecule does not pass the filter. This threshold was selected based on an equivalent analysis of the PDBbind 2020 data set,^55^ selecting the value for the 95th percentile of the energy ratios (Figure S4). In cases where the filtering pipeline results in few filtered compounds, this filter can be removed to increase the number of possible hits. Conformer generation is instead performed using only Fragmenstein (described below), which requires substantially greater compute time.

#### Filtering Compounds That Clash with the Protein Pocket

Compounds are filtered according to whether the merge fits the protein pocket. RDKit^44^ is used to calculate the protrusion volume between the merge and the protein (the proportion of the volume of the merge that protrudes from the protein). Molecules for which at least 16% of the volume clashes with the protein are removed. This threshold was set using analysis of Mpro crystallographic data. The clash distance between each compound and all protein structures was calculated, and the value for the 95th percentile was used to set the threshold (Figure S5).

#### Filtering Compounds Following Pose Generation with Fragmenstein

Poses are generated using a tool called Fragmenstein,^56^ which was developed as part of the COVID Moonshot project. Similar to the constrained embedding filter described above, Fragmenstein attempts to generate poses using the coordinates of the parent fragments, but is more computationally expensive and thus is used at the final stage of filtering. Fragmenstein generates poses by calculating the MCS and the positional overlap between the merge and the fragments and uses the crystallographic atom coordinates for placing the merge into the protein structure. Following placement, the conformation undergoes energy minimization using PyRosetta.^57^ Compounds are filtered for those for which Fragmenstein is able to generate a physically reasonable structure using the coordinates of both fragments. Other filters, including a combined RMSD with the fragments of <1 Å, a negative ΔΔ*G*, and the energy filter described above (to rule out unrealistic conformations), are also applied. A timeout was implemented of 10 min per molecule; molecules for which poses were not generated within this time limit were removed (this can occur for up to 10% of compounds entering the filter).

### Computational Workflow: Scoring and Analysis

Filtered compounds are subsequently scored using various metrics to allow comparison between the two techniques. Packages used for scoring are described below.

#### Prediction of Protein–Ligand Interactions

The protein–ligand interaction profiler (PLIP) was used for predicting protein–ligand interactions. PLIP calculates five types of interaction: hydrophobic contacts, π-stacking interactions (face-to-face or edge-to-face), hydrogen bonds (with the protein as a donor or acceptor), salt bridges (with the protein positively or negatively charged and with metal ions), and halogen bonds.^58^

The predicted interactions were compared with those of the parent fragments to discern whether the merge is able to replicate unique interactions made by each of the fragments (representing useful merges). In addition to comparing chemical diversity of the compound sets, we also compared functional diversity by looking at the interactions made by each compound set; the analysis adapts the methodology described in ref (59). Interaction diversity was analyzed for each target by selecting the minimal subset of filtered compounds that represent all possible interactions (compounds are ranked according to the amount of “information” recovered and are added until reaching a final set in which all possible interactions are recovered). This was repeated 100 times, shuffling each time (as multiple compounds will recover equivalent amounts of information). In each subset, the proportion of compounds that was proposed by either the Fragment Network or the similarity search was calculated to evaluate how diverse each compound set is with respect to diversity of interactions made.

#### Low-Dimensional Projections of Chemical Space

T-SNE plots were used to create low-dimensional representations of the compound data to allow easy visualization of chemical space.^60^ The default parameters set in scikit-learn (version 1.0.1)^61^ were used to generate the images, with the maximum number of iterations set at 3000.

### Retrospective Analysis Using Experimental Data

We provide details of two separate case studies using inhibitors designed against Mpro and *M. tuberculosis* transcriptional repressor protein EthR.^21^

#### Mpro

Existing experimental data consisting of IC_50_ values exist for Mpro and were used for retrospective analysis using a separate test set of fragments. We used data for compounds that were proposed and screened via the COVID Moonshot project and are available from the PostEra website.^10,62^ We ran an independent analysis for five fragments that were used as inspiration for the manual design of 24 compounds (under submission name TRY-UNI-714a760b), eight of which have recorded IC_50_ values in the low-to-medium micromolar range. This analysis was conducted independent of the main analysis, and thus, these five fragments are not included in the main test set described above. Furthermore, one of the inspirational fragments (fragment x1382-0A) is a covalent binder; however, the designed merges do not incorporate the chlorocetamide group and hence provide a useful test scenario for validating our method.

#### EthR

Nikifirov et al.^21^ describe a fragment-merging approach to designing *M. tuberculosis* EthR inhibitors using two fragment hits that were found to bind to the protein’s hydrophobic cavity in two different locations using X-ray crystallography (denoted compounds 1 and 2). The two fragments were used to propose three fragment merges based on the different arrangements in the cavity (compounds 3–5), of which compounds 4 and 5 were found to overlap with the volumes of their parent fragments. Several analogues of compound 5 were also synthesized (compounds 14–22) and were assayed using surface plasmon resonance (SPR) to give IC_50_ values. We use compounds 4, 5, and 14–22 to determine whether the Fragment Network is able to identify known binders or close analogues to known binders.

## Results

We tested the ability of the Fragment Network to identify merges using the results of crystallographic fragment screens against four targets: DPP11, PARP14, nsp13, and Mpro. Fragment hits against each target were selected, and pairs of fragments were enumerated for merging. We contrasted the results with that of a more standard fingerprint-based similarity search against the equivalent database of compounds. The resulting merges identified from both techniques were passed through a filtering pipeline, comprising both 2D and 3D filters, to prioritize the most promising molecules based on properties such as molecular descriptors, the ability to fit the protein pocket, and whether the compound is predicted to bind in such a way that maintains the orientation of the original fragments. Final poses were generated using Fragmenstein, and the resulting molecules were scored to allow comparison of performance.

### The Fragment Network Readily Identifies Pure Merges

For all targets, the Fragment Network yielded significant numbers of candidate merges that qualify as pure merges, as they could be placed in close agreement (<1 Å RMSD) with their parent fragments (Table 2). We differentiate between four types of merging opportunities based on the overlap between the parent fragments (Figure S6):

1. *Complete overlap merges* occur when most of the volume of one fragment overlaps with the other.
2. *Partial overlap by ring* occurs when only a ring overlaps between fragment pairs. These are the “classical” merges described in the literature and are akin to the ligand overlap required for molecular hybridization. In these cases, the merging process is relatively straightforward as there is a clear hypothesis for the connectivity of the final molecule.
3. *Partial overlap without ring* occurs when the overlap is some other substructure. In these cases, the connectivity of the final compound can be nonobvious.
4. *Nonoverlap of parent fragments* requires identifying linking opportunities, which were found in the PARP14 and Mpro data sets.

**Table 2:** Numbers of Filtered Compounds Identified Using a Fragment Network versus Similarity Search

	Number of hits	Search type^a^	Before filtering	After filtering	% filtered	Number of overlap	Complete overlap (%)	Partial overlap, ring (%)	Partial overlap, no ring (%)	No overlap (%)
DPP11	11	FN	22,903	198	0.9	8	0.0	29.4	70.6	NA
		SS	85,919	271	0.3		4.0	32.0	64.0	NA
PARP14	13	FN	48,320	70	0.1	0	0.0	18.3	45.1	36.6
		SS	78,116	56	0.1		16.1	16.1	37.5	30.4
nsp13	9	FN	36,239	503	1.4	1	0.4	99.6	0.0	NA
		SS	40,102	530	1.3		1.6	97.4	1.0	NA
Mpro	19	FN	109,012	952	0.9	4	2.5	6.8	49.6	41.2
		SS	169,424	918	0.5		71.0	7.1	12.5	9.4

a FN, Fragment Network; SS, similarity search.

**Figure 2:**
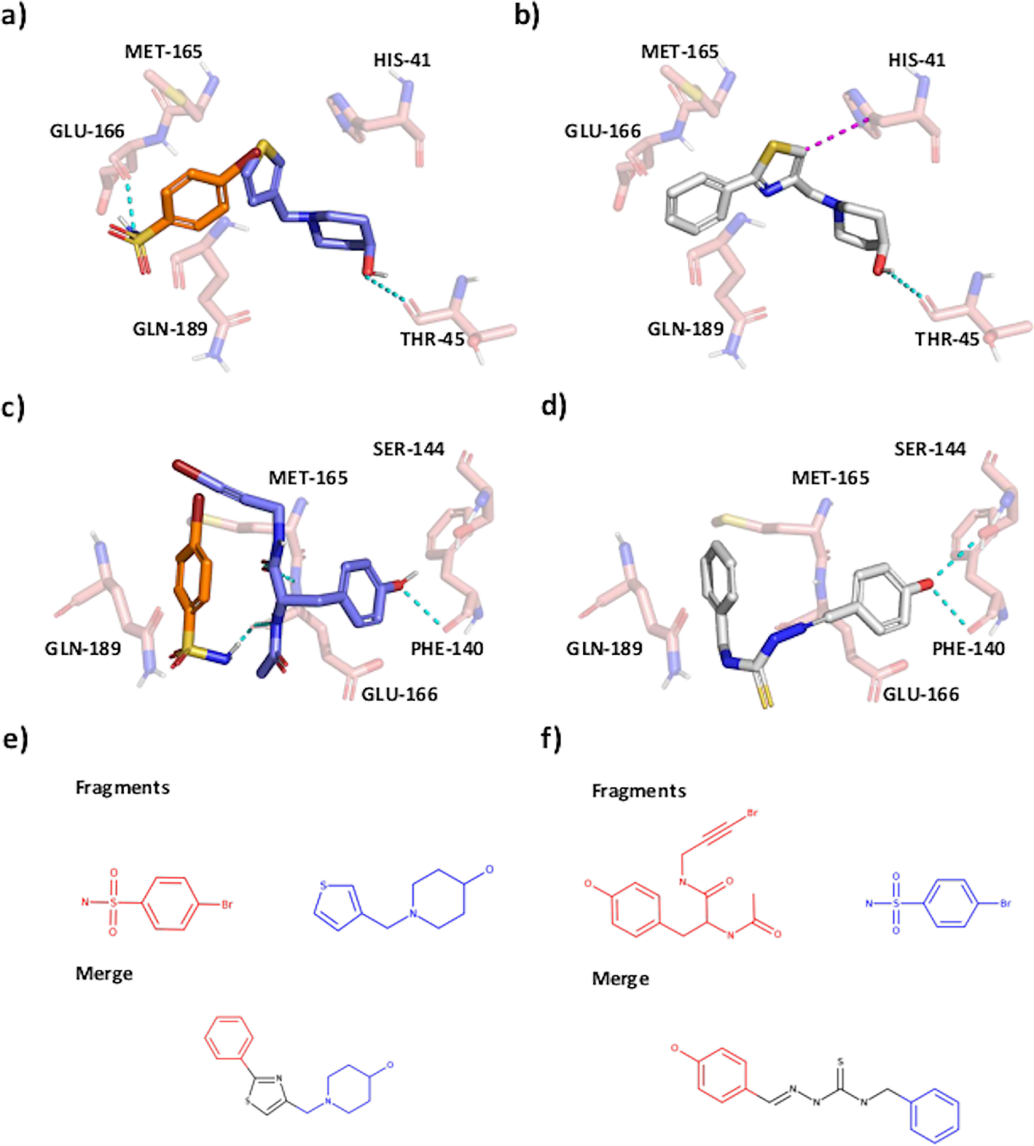
The Fragment Network identifies pure merges. (a) A fragment-merging opportunity for the main protease (Mpro) data set. Interactions are predicted using the protein–ligand interaction profiler (PLIP). Hydrogen bonds and π-stacking interactions are shown by cyan and magenta dotted lines, respectively. The PanDDA density is provided for the purple fragment in the Supporting Information (owing to the unusual conformation). (b) The linker-like merge (pose generated using Fragmenstein) joins substructures from partially overlapping fragments by a “linker-like” region, maintaining the hydrogen bond with THR-45; a change in orientation of the thiazole ring (with respect to the thiophene ring in the fragment) enables an additional π-stacking interaction with HIS-41. (c) A fragment-linking opportunity for Mpro. (d) The proposed compound maintains a hydrogen bond with PHE-140 and makes an additional bond with SER-144. It is worth noting that the linker group proposed by the Fragment Network is present in thioacetazone, an oral antibiotic. (e) The fragments and merge in a and b in 2D. (f) The fragments and merge in c and d in 2D.

Categories 3 and 4 can be considered “linker-like” merges, and for these, it tends to not be immediately evident which pieces of the parent fragments to incorporate into the final merge and how to do so in a way that will result in a physically reasonable structure that maintains the binding pose of the fragments. Nevertheless, for several such pairs, the Fragment Network identified how to join two distinct substructures of the parent fragments by a molecular linker, resulting in chemical series with diversity in the “linker” region, created by the intermediate optional hops in the database query (Figure 2; Figure S7).

The Fragment Network enables searches not just for merges but also linkers, by including fragment pairs up to 5 Å apart. Linking both fragments in their entirety is typically difficult, as it can lead to compounds that are neither purchasable nor synthetically accessible. Instead, our search links partial structures of each parent, thereby generating many candidates; for Mpro, it identified 419 compounds that are as much linkers as merges.

### Fragment Network and Similarity Searches Find Comparable Numbers of Compounds

The outputs of the Fragment Network and similarity searches are compared in Table 2. The totals show the number of unique compounds found across all pairs of fragments, even though the same candidate compound can be identified across multiple fragment pairs. All instances of each compound are filtered, as the 3D structures of the parent fragments may affect their chances of successful filtering. The results show that the techniques produce comparable numbers of filtered compounds across all targets, while the filtering efficiency differed substantially between targets, from 0.1% for PARP14 compounds to 1.3%–1.4% for nsp13 compounds.

**Figure 3:**
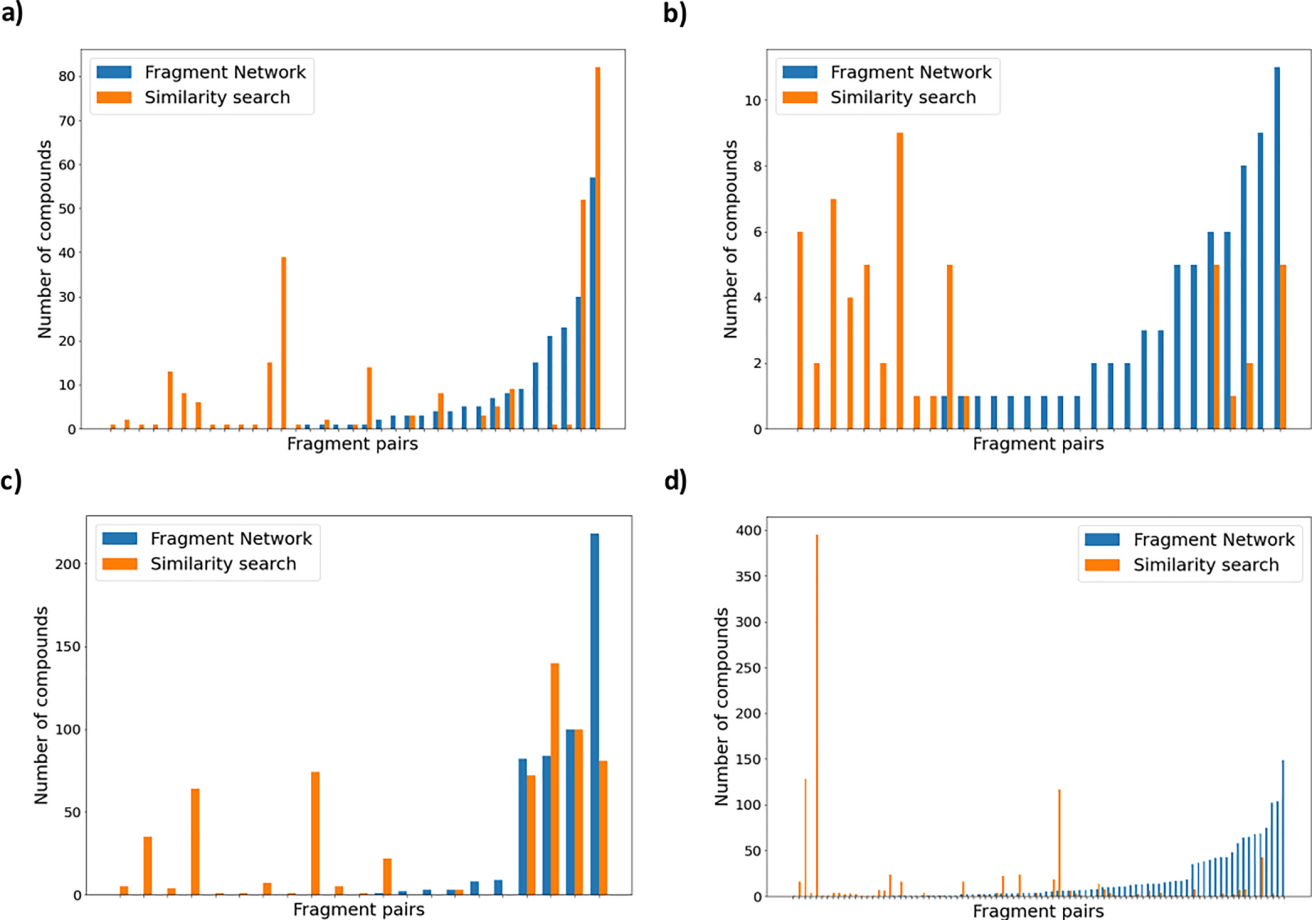
The Fragment Network and similarity searches identify filtered compounds for different fragment pairs. The numbers of filtered compounds for each fragment pair found using the Fragment Network (blue) or similarity search (orange) are shown across targets (a) dipeptidyl peptidase 11 (DPP11), (b) poly(ADP-ribose) polymerase 14, (PARP14), (c) nonstructural protein 13 (nsp13), and (d) main protease (Mpro). Only pairs that resulted in filtered compounds are shown. Pairs are ordered from right to left according to the number of Fragment Network compounds found. The data show that each search technique was able to identify filtered compounds for pairs where the other technique identified none.

Figure 3 shows the number of filtered compounds per pair using each technique, highlighting that this value is highly dependent on both the fragment pair and the technique used, with some pairs yielding no filtered merges from either technique. It was therefore not meaningful to compare scoring metrics between techniques, as there are few fragment pairs resulting in comparable numbers of filtered compounds for both techniques.

The number of merges found by the Fragment Network depends on several factors. First, highly connected seed fragments (typically smaller molecules that decompose into simple, common substructures) will result in more possible query paths and thus greater likelihood of finding merges that incorporate the substructure of interest. Second, a query path that involves an ubiquitous, promiscuous node (for example, if an optional hop yields a benzene) will pull out more paths, as these nodes typically make hundreds of thousands of connections. Third, during filtering, if the fragments in the pair are well-aligned and share overlapping substructures, placement of the merge is more likely to be successful. Fourth, if the query molecule is expanded by a common substructure, this will result in many paths. Specifically, the most common substructure used across all targets for expansion was a benzene ring, which is also a substructure for many of the catalogue compounds. For nsp13, 495 of the 510 (97%) filtered compounds (not accounting for redundancy) were identified by incorporating a benzene. However, the substructures incorporated for the other targets were more diverse, with benzene accounting for 37%–52% of expansions (Table S2).

The number of merges identified by the similarity search is affected by different factors; while it identifies comparable numbers of classical merges (category 2; Figure S8) to the Fragment Network, it is much less effective at identifying linkers (category 4), as evident for Mpro, where the similarity searches identify only 90 linker compounds, compared with 419 from the Fragment Network. Completely overlapping (category 1) merges also present a peculiar difficulty, since it is inherently more difficult to filter out compounds that represent expansions rather than true merges, as it is harder to codify the rules for merges that are maintaining bits of the parent fragments rather than substructures. For example, fragments x0195-0A and x0946-0A of the Mpro data set both share a phenylsulfonamide structure (Figure S9), for which the similarity search identified 395 filtered compounds. However, most of the merges were analogues, as they maintained the phenylsulfonamide but had no unique contributions from the remainder of the fragment structures.

### The Fragment Network and Similarity Search Identify Compounds from Distinct Areas of Chemical Space

There is very little overlap between the filtered compounds from the two search techniques, with a maximum of eight compounds found to be in common for DPP11 (Table 2). Figure 3 shows that each technique is able to identify merges for merge pairs for which the other technique identifies none, supporting the notion that these techniques are complementary and could be used in parallel to increase the potential resultant hit rate of a catalogue search (Table S3).

**Figure 4:**
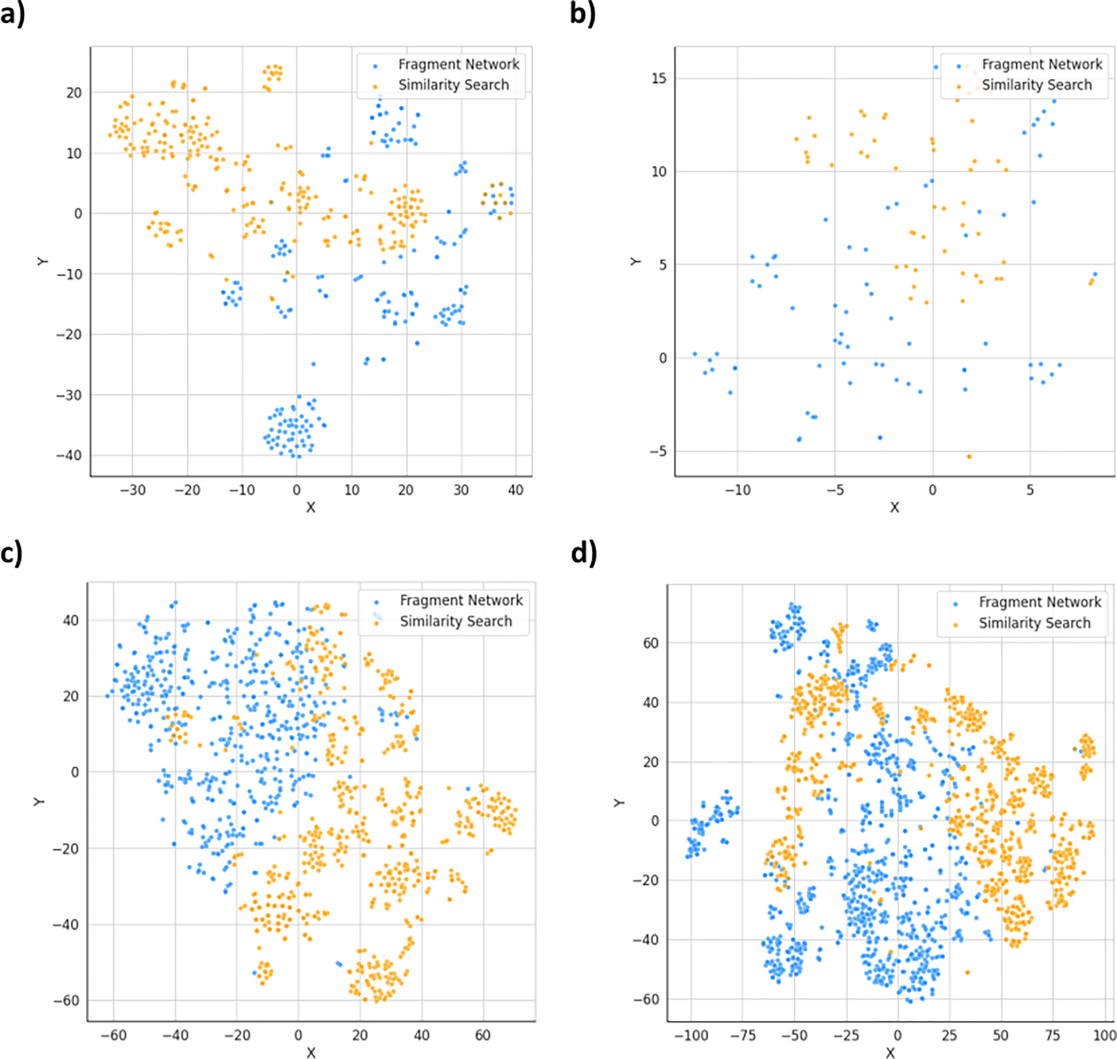
Fragment Network and similarity search-derived compound sets populate different regions of chemical space. The chemical space occupied by the filtered compound sets is projected into two dimensions using the T-SNE algorithm across targets (a) dipeptidyl peptidase 11 (DPP11), (b) poly(ADP-ribose) polymerase 14 (PARP14), (c) nonstructural protein 13 (nsp13), and (d) main protease (Mpro). Fragment Network compounds are shown in blue, and similarity search compounds are shown in orange. The two compound sets are shown to occupy distinct areas of chemical space.

The T-SNE projections in Figure 4 further demonstrate that these techniques operate in distinct areas of chemical space, as both techniques seem to be clustered in different regions and show little overlap. This is particularly apparent for nsp13 and Mpro, owing to the greater availability of data. Figure 4 also shows that the Fragment Network-filtered compounds show greater overall chemical diversity. The compounds were clustered using the Taylor–Butina clustering algorithm (using a Tanimoto distance threshold of 0.3),^63^ and the numbers of clusters containing only Fragment Network or similarity search compounds were counted. The Fragment Network was found to result in more clusters across all targets (Figure S11). The mean Tversky similarity between all filtered compounds and their parent fragments were also calculated (Figure S12); the Fragment Network compounds are far more dissimilar using these metrics and are able to access areas of chemical space not reached by the fingerprint-based similarity search, resulting in greater diversity in the compounds retrieved.

### Fragment Network and Similarity Searches Identify Compounds That Form Interactions with Residues Not Reached by the Other Technique

Interactions made by the merge compounds were predicted using PLIP. Across both the Fragment Network and similarity searches, the filtered compound sets were found to interact with the same or greater number of residues than the parent fragments used to create the respective merges. Furthermore, the Fragment Network and similarity search filtered compound sets were each found to make interactions with residues that the other compound set was not able to reach, in particular for DPP11 and PARP14; for nsp13, the Fragment Network reached residues not reached by similarity search, while the opposite was shown for Mpro (Figures S13–S16 and Table S4). We also calculated the number of merges that represent true merges in terms of residue-level interactions, meaning merges that are able to replicate unique interactions made by each of the parent fragments (after discounting interactions that are made by both of the parent fragments). The Fragment Network was more efficient for identifying this type of “true merge” for PARP14 and Mpro, while the similarity search performed better for nsp13 and DPP11 (Table S5). This suggests that the two techniques may perform better for different targets.

In order to explore the concept of “interaction diversity”, whereby compounds are selected according to the diversity in the interactions made (functional diversity) rather than their chemical diversity, we performed an analysis to identify the smallest compound sets for each target that make all possible interactions with the protein. This is done across both the Fragment Network and similarity search-identified compounds. In the final compound sets, we found that compounds from both search approaches were represented, again supporting the idea that the two techniques are complementary (Table 3). For the purposes of identifying compounds for purchase and further screening, we argue that these results support performing both techniques in parallel for catalogue search, expanding the set from which to identify the most useful purchase list for further screening and informing SAR.

**Table 3:** Composition of Functionally Diverse Subsets Identified from Filtered Compound Sets^a^

Target	Fragment Network compounds (%)	Similarity search compounds (%)
DPP11	39.7 ± 5.0	60.3 ± 5.0
PARP14	40.3 ± 5.4	59.7 ± 5.4
nsp13	74.0 ± 2.8	26.0 ± 2.8
Mpro	30.5 ± 4.3	69.5 ± 4.3

a Mean and standard deviation are provided.

### Efficiency of the Filtering Pipeline

A series of 2D and 3D filters was implemented to remove compounds that lack desired molecular properties, that may not represent true merges, that do not fit the protein pocket and that are unable to replicate the binding pose of the original fragments. Table S6 shows the percentages of compounds that are removed by each step in the filtering pipeline (as both the percentage of total compounds and the percentage of compounds entering the filter). The Fragment Network search typically identifies larger molecules and molecules that contain long linker regions containing many rotatable or consecutive nonring bonds, while the similarity search compounds are typically more conservative with regard to their size. The constrained embedding filter removes a greater proportion of similarity search compounds compared with Fragment Network compounds. A possible explanation for this is that the Fragment Network is more likely to preserve exact substructures of the parent fragments, and thus, the MCS calculation used to extract the atomic coordinates of atoms to use as the embedding template is more likely to yield a greater number of atoms. Across both techniques, the step involving pose generation with Fragmenstein and filtering based on the resulting conformations is the most restrictive filter. We would expect this step to be the most conservative, as it is the most accurate and computationally intensive filter in our pipeline for determining whether the molecule can adopt a sensible binding pose that mirrors that of the parent fragments.

### The Fragment Network Has Efficiency Benefits over Similarity Search

The Fragment Network search is more computationally efficient than the similarity search. The Fragment Network querying was run single-threaded and takes an average of 2–14 min (dependent on the target) per fragment pair. The similarity search was run on 16 CPUs and takes 2–3 min per fragment pair, requiring up to 40 min in total of CPU time. The time required for filtering all merges for a target ranged from 5.2–75.9 CPU days (this value varies considerably depending on the target and search technique). Timings for filtering and placement of molecules using Fragmenstein are provided in Table S7. Conformer generation using Fragmenstein represents the most computationally expensive step; however, this step could easily be replaced with other placement tools such as constrained docking programs to speed up the pipeline if desired.

In comparison, building the Fragment Network database is a more computationally expensive procedure. To perform the fragmentation and processing of five million molecules for input into the Network requires on average 49 CPU days (this value is highly variable and dependent on molecular complexity). However, given the composition of the database and ability to represent entire catalogues, this is a rare operation to undertake as the overall chemical space of the catalogues will not change drastically over short time scales. This precomputation also allows a more thorough exploration of chemical space than possible using a similarity search alone. Moreover, the addition of new molecules (as catalogues are updated) will not require the reprocessing of all molecules and thus would be substantially less intensive. For example, we could perform the fragmentation of approximately 700,000 molecules within 7 CPU days and thus should be able to perform updates at a rate that exceeds the rate of growth of any commercial catalogue.

### Retrospective Analysis Using Experimental Data

#### Mpro

To test whether the Fragment Network is able to identify true binders, a retrospective analysis was performed using data from the public COVID Moonshot.^10^ A set of five fragments were originally used to propose the manually designed TRY-UNI-714a760b series of fragment merges, which successfully achieved on-scale potency for several compounds. We queried the Fragment Network with the same fragments and looked for TRY-UNI-714a760b compounds in the results. Three of the five fragments are either highly similar or represent substructures of each other, and thus, pairs between these fragments were removed, resulting in seven pairs of fragments for querying. We also compared our proposed merges against all other compounds with experimental data but without a covalent warhead. This resulted in a total of 1247 compounds with an IC_50_ value of <99 μM against Mpro recorded by either a fluorescence or RapidFire mass spectrometry (RF-MS) assay.

**Figure 5:**
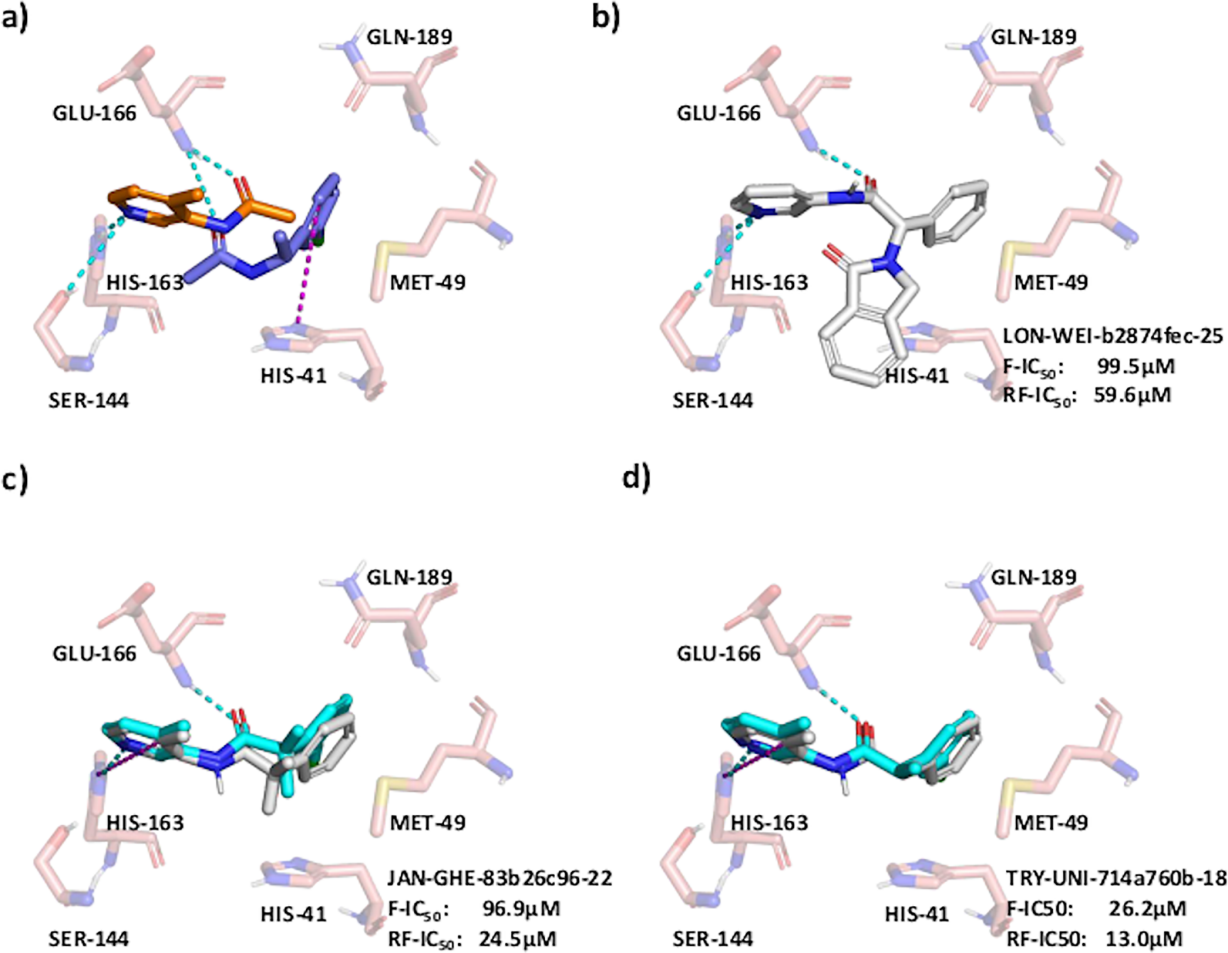
The Fragment Network identifies a known binder against Mpro. A Fragment Network search using (a) two fragment hits against the SARS-CoV-2 main protease (Mpro) identifies, (b) a known binder against Mpro (LON-WEI-b2874fec-25; RapidFire mass spectrometry (RF-MS) IC_50_ value of 59.6 μM) and similar compounds to known binders, (c) JAN-GHE-83b26c96–22 (fluorescence and RF-MS IC_50_ values of 96.9 μM and 24.5 μM), and (d) TRY-UNI-714a760b-18 (fluorescence and RF-MS IC_50_ values of 26.2 μM and 13.0 μM). Fragmenstein-predicted merge poses are shown in white, and crystal poses are in cyan. Interactions are predicted using the protein–ligand interaction profiler (PLIP), and key interaction residues are shown. Hydrogen bonds are shown in cyan, and π-stacking interactions are shown in magenta.

After filtering, the Fragment Network did identify one known binder, along with compounds highly similar to several other known binders (Figure 5; Figure S17). The direct match, LON-WEI-b2874fec-25, has an RF-MS IC_50_ value of 59.6 μM; between the filtered compounds and the known binders, there were 13 pairs with Tanimoto similarity of >0.6, including TRY-UNI-714a760b-18 and TRY-UNI-714a760b-22 (Tanimoto similarities of 0.73 and 0.68, respectively; Figure 5d). Where available, the Fragmenstein-generated poses closely mimic the crystal poses. Compound TRY-UNI-714a760b-16 was also identified, which does not have an IC_50_ value.

Two additional known binders (JAN-GHE-83b26c96-22 and TRY-UNI-714a760b-18) were retrieved by mitigating the limitation of the current implementation of the Fragment Network described in the section Search by Fragment Network Query, which necessitates expansions to be made with substructures containing at least three carbons. When we performed R-group expansions on the retrieved analogues, using substituents from the original fragments, the additional binders were identified through the addition of a chlorine atom (Figures S18 and 19).

In comparison, the similarity search after filtering identified three of the retrieved analogues (JAN-GHE-83b26c96-22, TRY-UNI-714a760b-18, and TRY-UNI-714a760b-22). However, the similarity search did not identify the Fragment Network match LON-WEI-b2874fec-25, due to the low Tversky similarity between this compound and the parent fragments. This further supports the complementary nature of these two search techniques.

#### EthR

We perform an additional analysis using two fragments found to bind to EthR (each fragment binds in two different conformations). As the two fragments already exist in the database, the Fragment Network search was performed using the two fragments, and filtering was repeated using all four pairs of the different fragment conformations. This resulted in 10 filtered compounds (an example is shown in Figure S20). While compound 4 was retrieved from the database, it was ruled out during filtering using our stringent default filtering parameters. As compound 4 was removed by the constrained embedding filter (a possible explanation for this is given below), we also tried relaxing the filtering pipeline by only using Fragmenstein for conformer generation, which increased the number of filtered compounds to 148.

**Figure 6:**
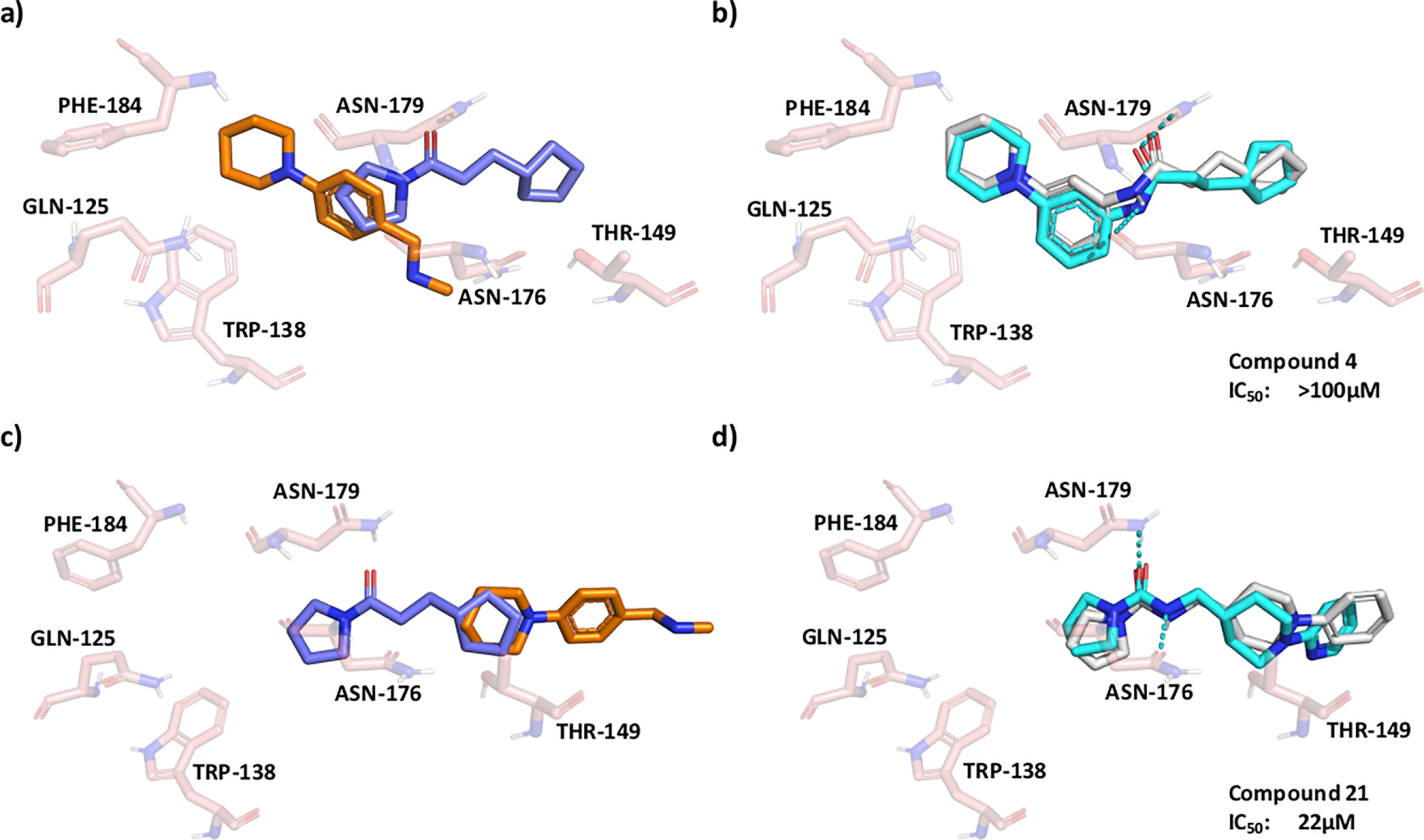
The Fragment Network identifies a known binder against EthR. A Fragment Network search using (a) two fragment hits against (which each bind in two different positions) *Mycobacterium tuberculosis* transcriptional repressor protein EthR identifies (b) a known binder (compound 4; IC_50_ value of >100 μM). (c) An alternative crystallographic arrangement of the equivalent fragments identifies (d) a similar compound to a known binder, compound 21 (IC_50_ value of 22 μM). Fragmenstein-predicted merge poses are shown in white, and crystal poses are in cyan. Hydrogen bonds are shown in cyan.

Following this change, the Fragment Network was able to successfully identify compound 4 after filtering (Figure 6). This exemplifies the benefit of implementing a flexible filtering pipeline that can be tailored according to the features of the test system. While the compound has weak potency, with an IC_50_ value of >100 μM, the Fragmenstein-predicted pose shows good overlap with the crystal pose. However, the change in orientation of the five-membered ring between the two structures (and with respect to the original fragment) is likely the reason for the initial failure of the constrained embedding filter, as RDKit was not able to generate a conformation that maintains the orientation of this ring in a physically reasonable way. While the manually designed compound 5 is not present in the network, the Fragment Network identified several compounds that were highly similar to analogues of compound 5, shown in Figure S21, with SPR IC_50_ values ranging between 2–25 μM. An example is shown in Figure 6d, for which the Fragmenstein-predicted pose again well reflects that of the original crystal pose. Moreover, the two compounds differ by a single nitrogen atom (which is not present in the original fragment and could not be expected to be identified by the Fragment Network pure merge search).

Collectively, these results provide validation for the use of the Fragment Network to progress crystallographic fragment hits to compounds with on-scale potencies in multiple systems.

## Discussion

We have illustrated the use of the Fragment Network, a graph database containing compounds from commercial catalogues, for finding fragment merges and show that our method can expand the scope of a traditional similarity-based catalogue search and increase the yield of potential follow-ups. We contrast the use of the Fragment Network and a fingerprint-based similarity search to find merges using the results of crystallographic fragment screens. The results support the idea that these two techniques are complementary and should be used in parallel, in that they are able to identify merges for pairs of fragments that are not represented by the other. They show little overlap in the compounds identified and chemical space in which they operate, and they are able to identify compounds that form interactions not represented by the other. In particular, the Fragment Network is well-suited to identifying what we refer to as pure merges, in other words, compounds that incorporate exact substructures of the parent fragments. The Fragment Network exploits a different type of similarity that is able to preserve substructures of the original fragments but that may appear dissimilar using a fingerprint-based metric. This also demonstrates usefulness beyond that of classical molecular hybridization techniques, which require overlapping substructures to exist between the set of input molecules.

The ability of the Fragment Network to identify known inhibitors of Mpro and EthR and highly similar compounds to inhibitors predicted to bind in the same orientation provides initial validation and supports the use of this approach in the FBDD pipeline. In practice, the types of filter used and the thresholds set will be dependent on the application and target. The pipeline has therefore been built with the flexibility to enable users to select which filters and scoring metrics to use and to set desired parameters and thresholds.

The nature of the pure merges identified has some repercussions; while the variation added to the molecule may not necessarily be better for maintaining all possible fragment interactions, we would hope that the preservation of exact substructures may result in the merges being more likely to mimic the original binding pose of the fragments.

Regarding efficiency, one of the main advantages of the Fragment Network approach is the reduced compute time required to run a search. As the size of commercial catalogues is ever-expanding and new approaches are needed for managing the vast availability of compound data, efficient search techniques represent an important avenue of research. Search techniques such as these could become especially relevant for large virtual libraries with increasing coverage of chemical space.

Future work could improve the efficiency of the search by limiting the substructures used in the expansion to those that are responsible for an interaction that we want to preserve in the final merge; similarly, we could ensure that the seed fragment is not reduced down to a substructure that is also not responsible for any interactions. Moreover, we acknowledge that a limitation of the pipeline is the ability to incorporate substituents from the parent fragments due to the limitation imposed on substructures for expansion of containing at least three carbon atoms. Because of the lack of atom mapping between nodes, it is not feasible to perform multiple expansions using more than one substructure from the original fragment while specifying where on the molecule the substituent should be added. Atom mapping is a feature we hope to implement in future iterations of the network, which will allow greater control over where substructures are added to the final merge.

## Conclusions

Currently, there are limited available *in silico* approaches for identifying fragment merges and, in particular, those that identify compounds that are synthetically accessible. We demonstrate the application of a graph database for identifying commercially available fragment merges that will allow rapid follow-up and progression of hits in FBDD campaigns.

Use of the Fragment Network has proved to be an effective way to improve the yield and potential hit rate of a catalogue search and has demonstrated itself to be complementary to a classical search using a fingerprint-based similarity metric. The network was able to successfully identify known inhibitors against Mpro and EthR, the former with micromolar activity. The results reported here support the use of the two techniques in parallel, with the aim of selecting the optimal compound subset for purchase from all enumerated compounds for further assaying and informing the next iteration of synthesis.

## Data Availability

The code to run the querying and filtering pipeline is publicly available through https://github.com/oxpig/fragment_network_merges. The code to generate the Fragment Network is available at https://github.com/InformaticsMatters/fragmentor. The query data retrieved from the database searches and the filtered compounds are available from https://zenodo.org/record/7957805. Access to the current snapshot of the database can be provided upon request.

## Supporting Information Available

Figures and tables referenced in the text (PDF)
