# Supplementary material for: Fragment Merging Using a Graph Database Samples Different Catalogue Space than Similarity Search
Source: J Chem Inf Model. 2023 May 25;63(11):3423–37. doi: 10.1021/acs.jcim.3c00276 (PMC10268959; doi:10.1021/acs.jcim.3c00276)
Supplement: Supplementary file 1 — ci3c00276_si_001.pdf [file ci3c00276_si_001.pdf]

Supplementary information

Fragment merging using a graph database samples different catalogue space than similarity search

Stephanie Wills[1,2], Ruben Sanchez-Garcia[1,2], Tim Dudgeon[3], Stephen D. Roughley[4], Andy Merritt[5], Roderick E. Hubbard[4], James Davidson[4], Frank von Delft[2,6,7,8], and Charlotte M. Deane[*,1]

[1]Department of Statistics, University of Oxford, Oxford, OX1 3LB, UK.
[2]Centre for Medicines Discovery, University of Oxford, Oxford, OX3 7DQ, UK.
[3]LifeArc, Lynton House, 7–12 Tavistock Square, London, WC1H 9LT, UK.
[4]Vernalis (R&D) Limited, Granta Park, Great Abington, Cambridge, CB21 6GB, UK.
[5]Informatics Matters Ltd., Perch Coworking, Franklins House, Bicester, OX26 6JU, UK.
[6]Diamond Light Source, Didcot, OX11 0DE, UK.
[7]Research Complex at Harwell Harwell Science and Innovation Campus, Didcot, OX11 0FA, UK.
[8]Department of Biochemistry, University of Johannesburg, Auckland Park 2006, South Africa.
[*]deane@stats.ox.ac.uk

Table S1: Fragments used for analysis

Target	Fragments
DPP11	x0267_0A, x0230_1A, x0208_0A, x0228_0A, x0199_0A, x0346_0A, x0051_0A, x0115_0A x0083_0A, x0056_0A, x0087_0A
PARP14	x0161_1, x0238_1, x0266_1, x0315_1, x0324_1, x0334_1, x0412_1, x0457_1, x0473_1 x0505_1, x0590_1, x0637_1, x0712_1
nsp13	x0034_0B, x0176_0B, x0183_0B, x0208_0A, x0212_0B, x0246_0B, x0276_0B, x0311_0B x0438_0B
Mpro	x0354_0A, x0426_0A, x0104_0A, x0195_0A, x0072_0A, x0305_0A, x0161_0A x1077_0A, x0874_0A, x1249_0A, x0991_0A, x2193_0A, x1093_0A, x0946_0A, x0967_0A x0395_0A, x0387_0A, x0540_0A, x0397_0A
Mpro (case study)	x0434_0A, x0107_0A, x0678_0A, x0995_0A, x1382_0A

Crystal complexes are available to download from the Fragalysis platform:
`https://fragalysis.diamond.ac.uk/viewer/react/landing`

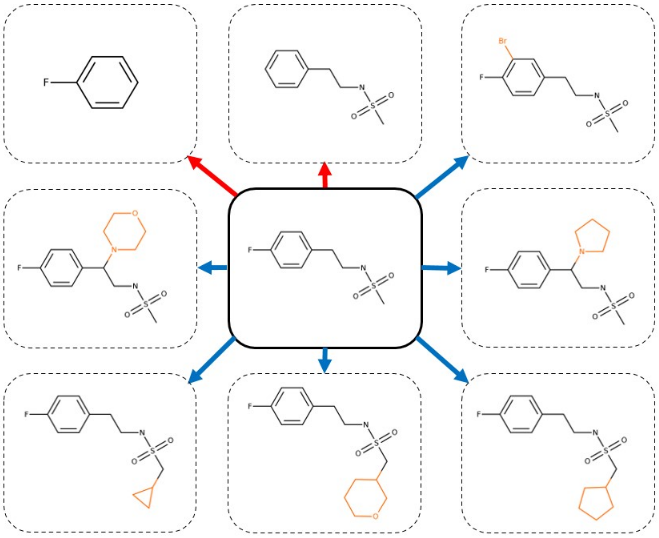

Figure S1: **Transformations between nodes in the Fragment Network.** Edges in the Fragment Network denote transformations in which a contraction (red arrows) or expansion (blue arrows) can be made, whereby a ring, linker or substituent is lost or gained. Example transformations are shown for a hit against non-structural protein 13 (nsp13; fragment x0276_0B). The substructure lost or gained during the transformation is recorded in the edge label.

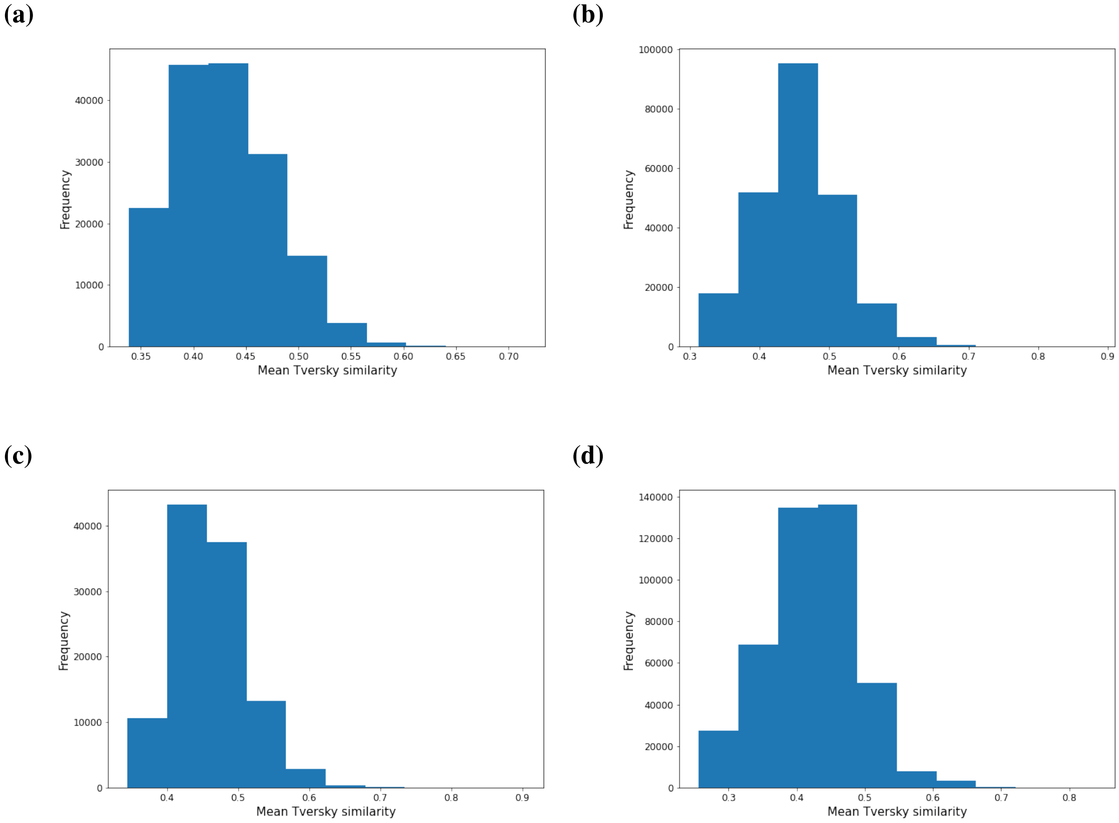

Figure S2: **The mean Tversky similarities between unfiltered merges and their parent fragments found using similarity search.** The mean Tversky similarity between similarity search-identified merges (before removing those with Tversky <0.4 and before entering the filtering pipeline) and their parent fragments are shown for targets (**a**) dipeptidyl peptidase 11 (DPP11), (**b**) poly(ADP-ribose) polymerase 14, (PARP14) (**c**) non-structural protein 13 (nsp13) and (**d**) main protease (Mpro).

Table S2: Substructures used in the expansion of Fragment Network-filtered compounds.

Target	Substructure SMILES	Frequency
DPP11	[*]c1ccccc1	83
	[*]N1CCOCC1	55
	[*]c1ccco1	26
	[*]C1CC1	22
	[*]N1CCCC1	12
	[*]c1ccncc1	3
	[*]c1ccoc1	2
	[*]N1CCc2ccccc2C1	1
PARP14A	[*]c1ccccc1	26
	CCC[*]	18
	[*]C1CCCCC1	9
	[*]n1cccn1	6
	[*]c1ccco1	5
	[*]c1ncccn1	3
	[*]c1ccc2ccccc2n1	2
	[*]c1nc2ccccc2[nH]1	1
	[*]c1cn2ccccc2n1	1
nsp13	[*]c1ccccc1	495
	[*]C1CNC1	11
	[*]N1CCC1	4
Mpro	[*]c1ccccc1	531
	[*]c1ccccn1	146
	[*]c1cccnc1	95
	[*]N1CCOCC1	85
	[*]N1CCCCC1	67
	[*]c1ccncc1	25
	[*]C1CC1	21
	[*]c1ccsc1	19
	[*]N1CCCc2ccccc21	15
	[*]C1CCNCC1	5
	[*]C1CCCCC1	4
	[*]N1CCCOCC1	3
	[*]c1c[nH]c2ncccc12	1
	[*]N1CCNCC1	1

[*] denotes the attachment point in the SMILES.

Table S3: The number of pairs represented by Fragment Network and similarity search filtered compounds.

	Number of pairs represented by each technique		
Target	Fragment Network only	Similarity search only	Both
DPP11	9	14	12
PARP14	15	9	6
nsp13	4	11	6
Mpro	29	18	36

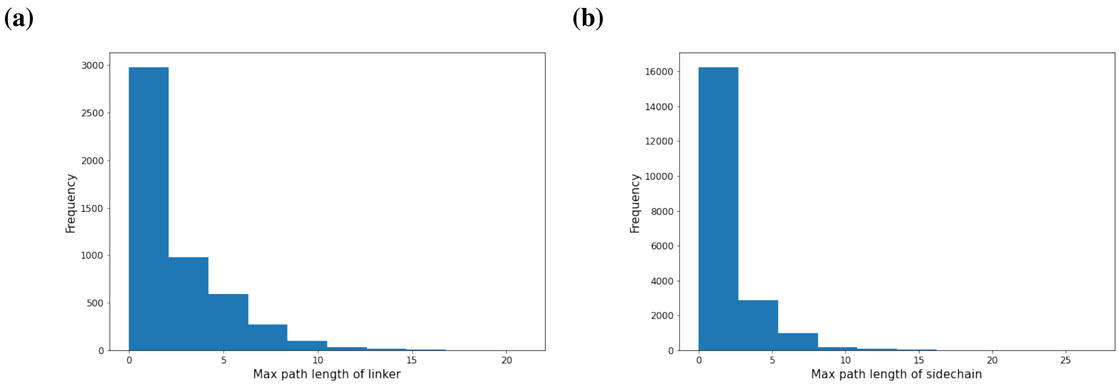

Figure S3: **The max linker and sidechain lengths found in ChEMBL drug molecules.** The max path length for (**a**) linkers, which join two rings, and (**b**) sidechains, which are attached to a single ring, was recorded for ChEMBL drug molecules (CHEMBL29; after applying Lipinski filters and a maximum rotatable bond limit of 10). The 95th percentiles were used to set thresholds for the filter.

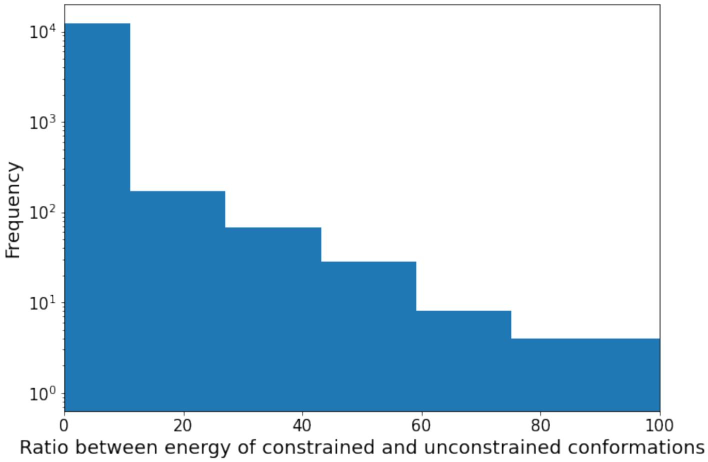

Figure S4: **Ratio between constrained and unconstrained conformations for PDBbind 2020 ligands.** The ratio between the energies of PDBbind ligands and 50 unconstrained conformers was calculated and plotted using a log scale.

Table S4: The number of interaction residues reached by Fragment Network and similarity search filtered compounds.

	Number of interactions reached by compound set						
Target	FN fragments	SS fragments	FN merges	SS merges	FN merges only	SS merges only	Both merges
DPP11	7	9	14	16	3	5	11
PARP14	10	10	10	14	1	5	9
nsp13	11	11	28	20	8	0	20
Mpro	20	18	25	32	0	7	25

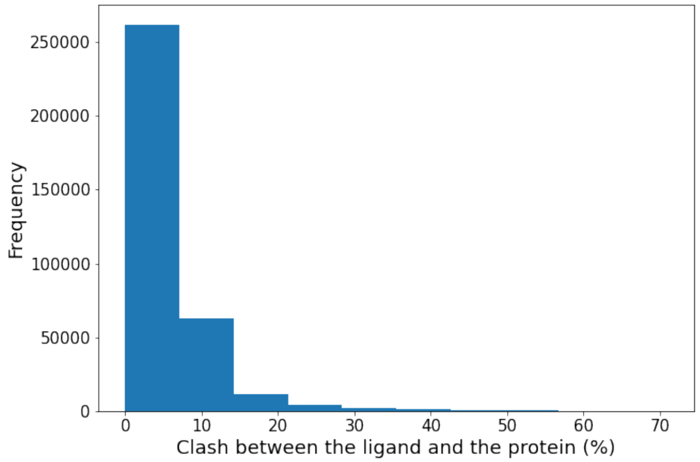

Figure S5: **Clash between Mpro ligands and all protein structures.** The protrusion between main protease (Mpro) ligands (available in Fragalysis) and all protein structures was calculated. The clash between all structures is shown.

Table S5: The number of 'true merges' found where both fragments contribute a unique interaction type with a specific residue to the final merge.

	Fragment Network			Similarity search		
Target	True merges	Possible true merges	Efficiency (%)	True merges	Possible true merges	Efficiency (%)
DPP11	32	203	15.8	88	272	32.4
PARP14	45	71	63.4	26	56	46.4
nsp13	82	509	16.1	142	616	23.1
Mpro	513	1,017	50.4	128	832	15.4

(a) (b) (c) (d)

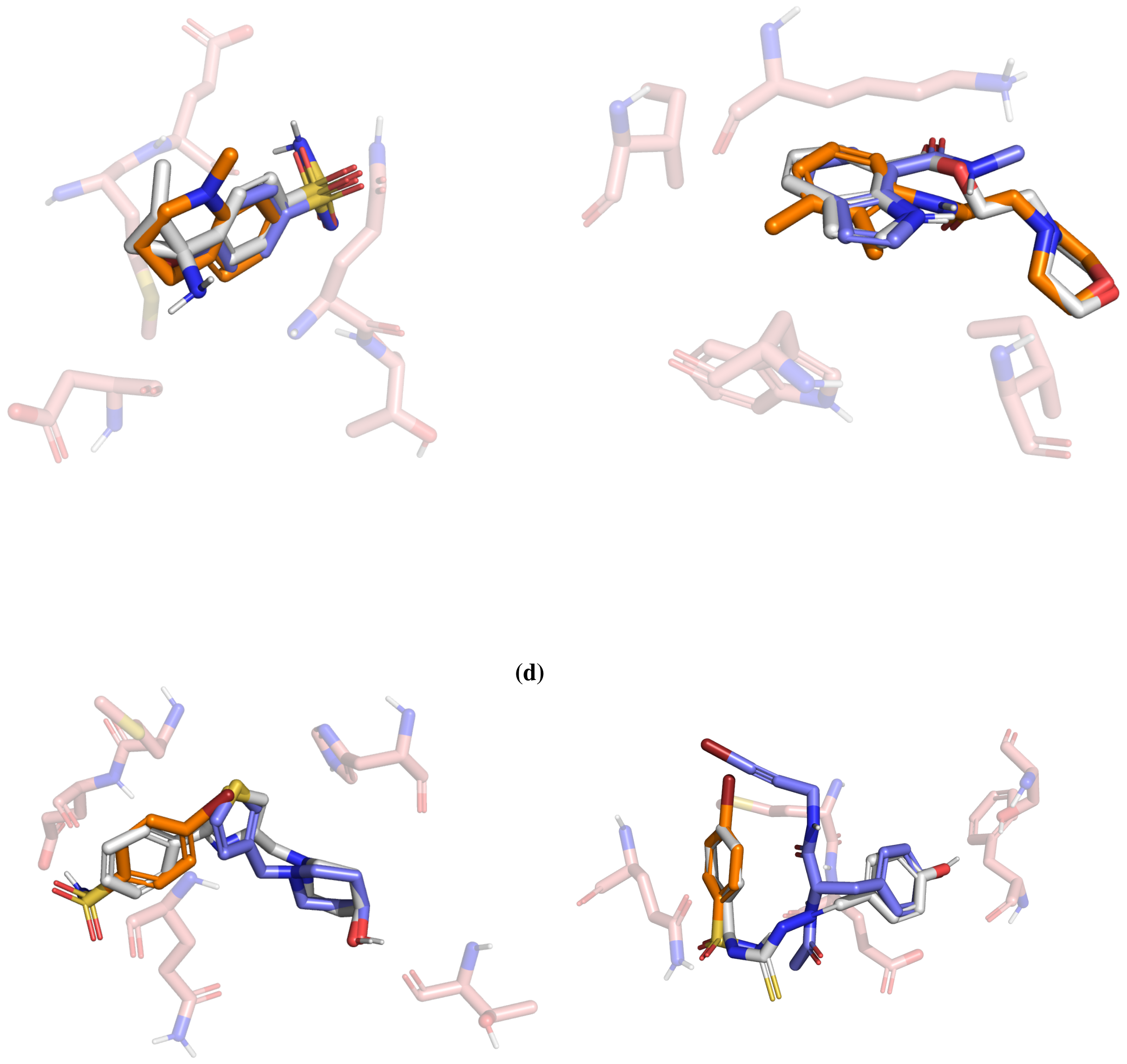

Figure S6: **Types of merging opportunity.** We differentiate between four different types of merging opportunity depending on the overlap between the parent fragments (shown in purple and orange): (**a**) *complete overlap merges*, whereby the majority of the volume of one fragment overlaps with the other; (**b**) *partial overlap by ring*, which are fragments that share an overlapping ring structure and represent the classical merges seen in the literature; (**c**) *partial overlap without ring*, whereby the overlapping fragments do not share an overlapping ring structure; and (**d**) *non overlap of parent fragments*, which represent linking opportunities.

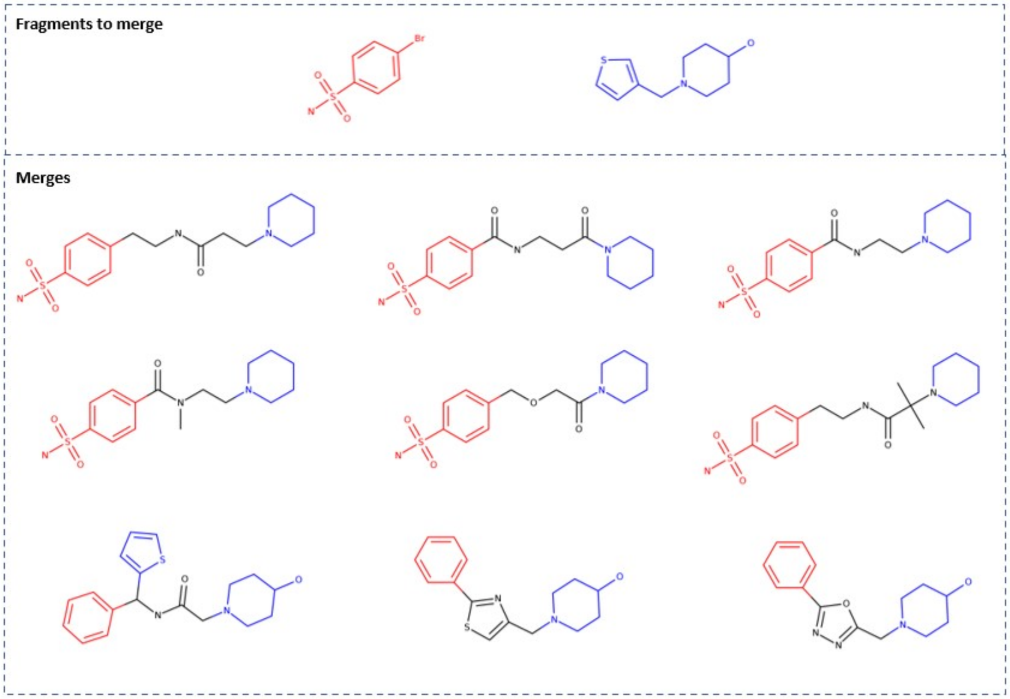

Figure S7: **Example linker-like merges.** Example ‘linker-like’ merges for two fragments found to bind to the main protease (Mpro). Diversity is generated in the ‘linker’ region joining the two substructures from the parent fragments.

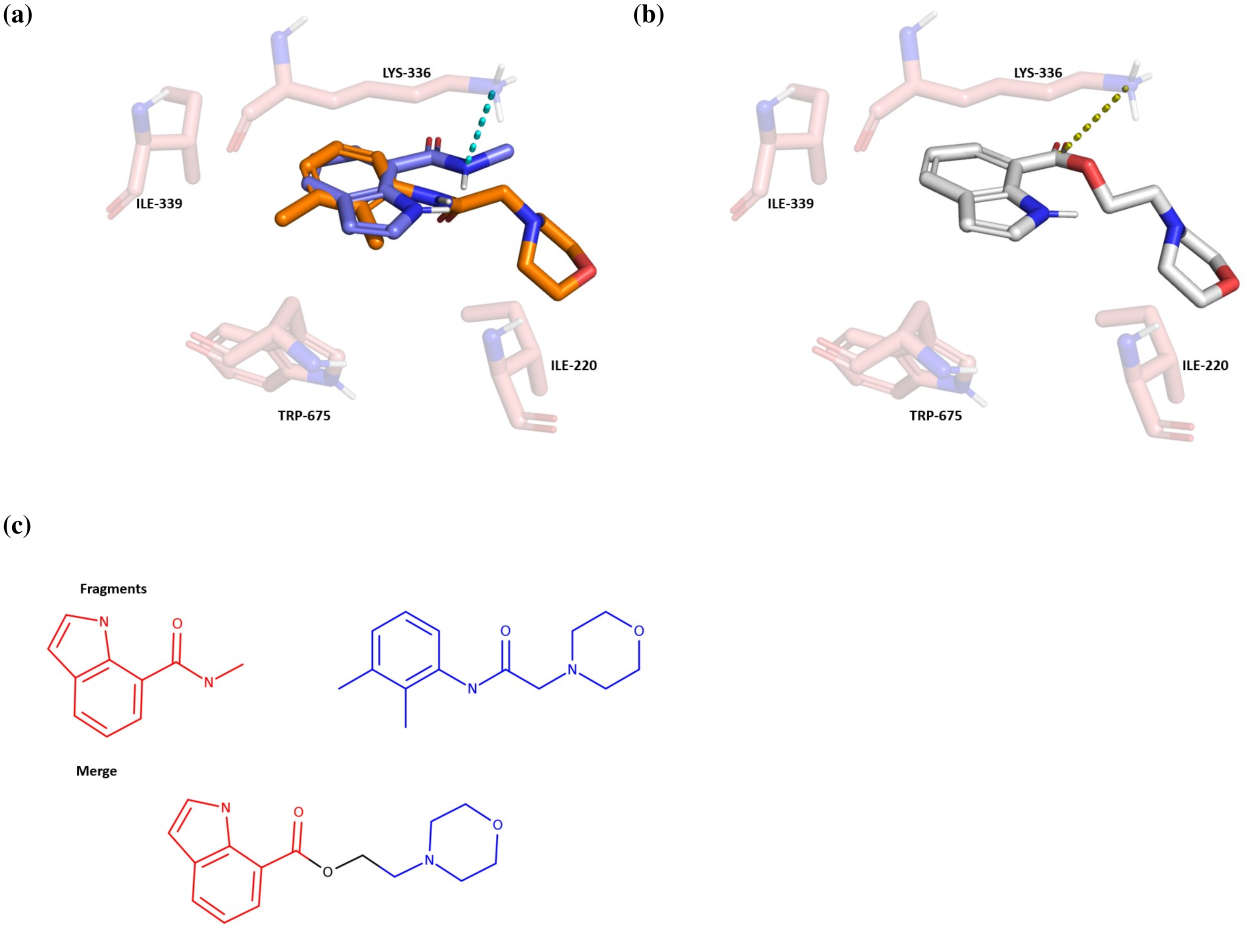

Figure S8: **An example classical merge.** (**a**) The crystal structures of two parent fragments that represent a 'classical merge' for dipeptidyl peptidase 11 (DPP11), whereby the two fragments show an overlapping ring and the connectivity of the final compound is obvious. (**b**) The orientation of the merge (white) generated using Fragmenstein. Interactions are predicted using the protein–ligand interaction profiler (PLIP) and key interaction residues are shown. A salt bridge is depicted using a yellow dotted line. (**c**) The fragments and merge in 2D; colours indicate the substructures used in forming the merge.

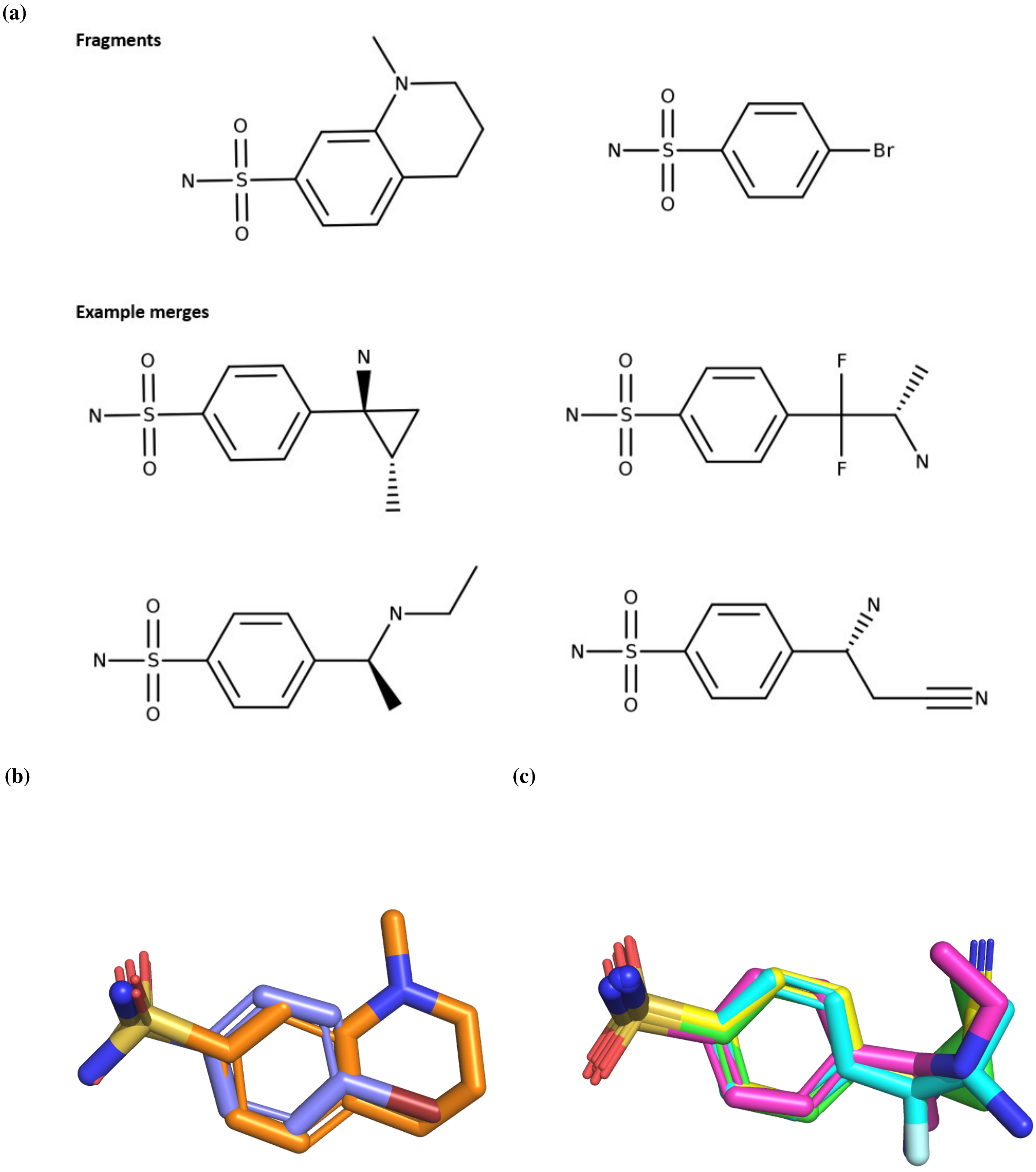

Figure S9: **Non-useful merges identified using similarity search.** (**a**) Example compounds identified using similarity search for two fragments (x0195-0A and x0946-0A) against the main protease (Mpro). The compounds do not represent useful merges as they do not incorporate unique substructures from both fragments. Crystal and Fragmenstein-predicted poses are shown of the fragments (**b**) and the merges (**c**), respectively.

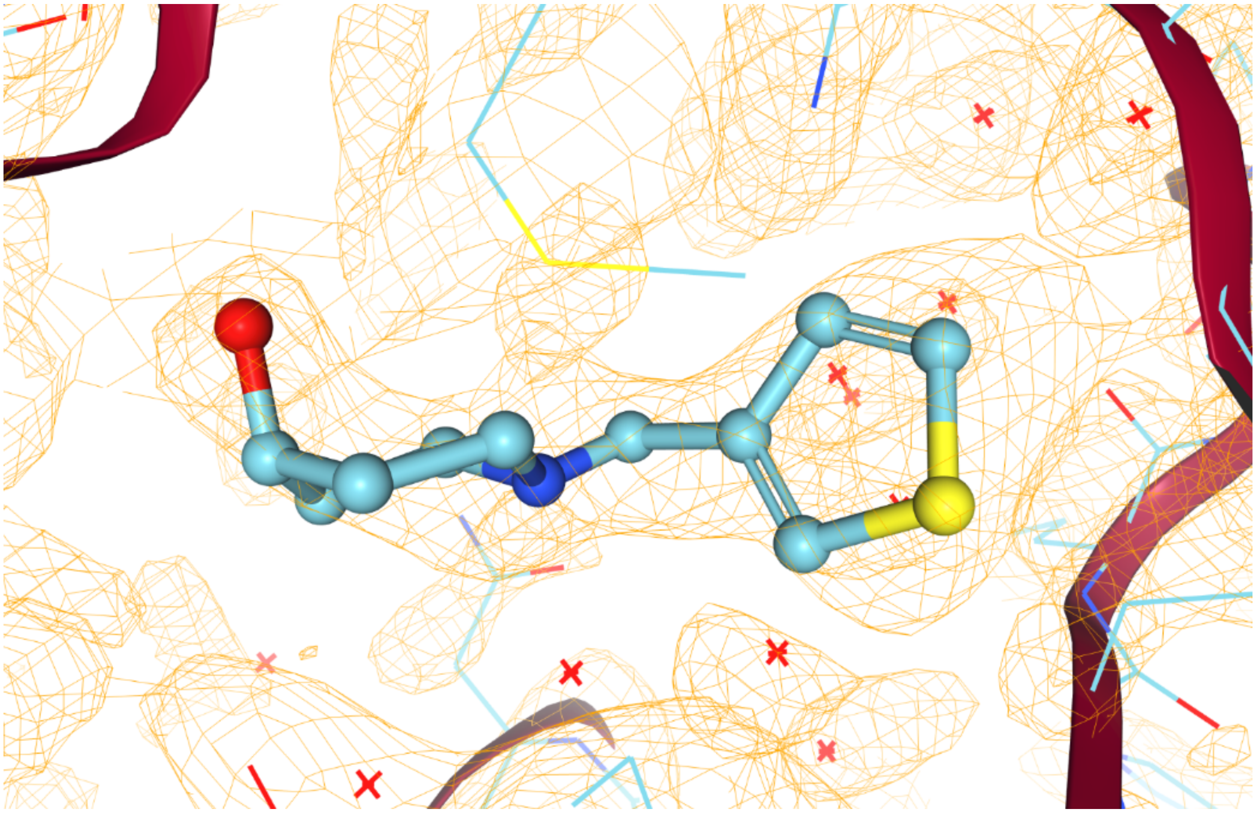

Figure S10: **PanDDA's density for Mpro fragment x0387_0A.** Due to the unusual conformation of this fragment, we provide a snapshot of the PanDDA event map, which supports the placment of the hydroxyl group. The Fragment Network merging pipeline proposes merges that are faithful to the conformation of the original fragments given the atomic coordinates that are provided as input.

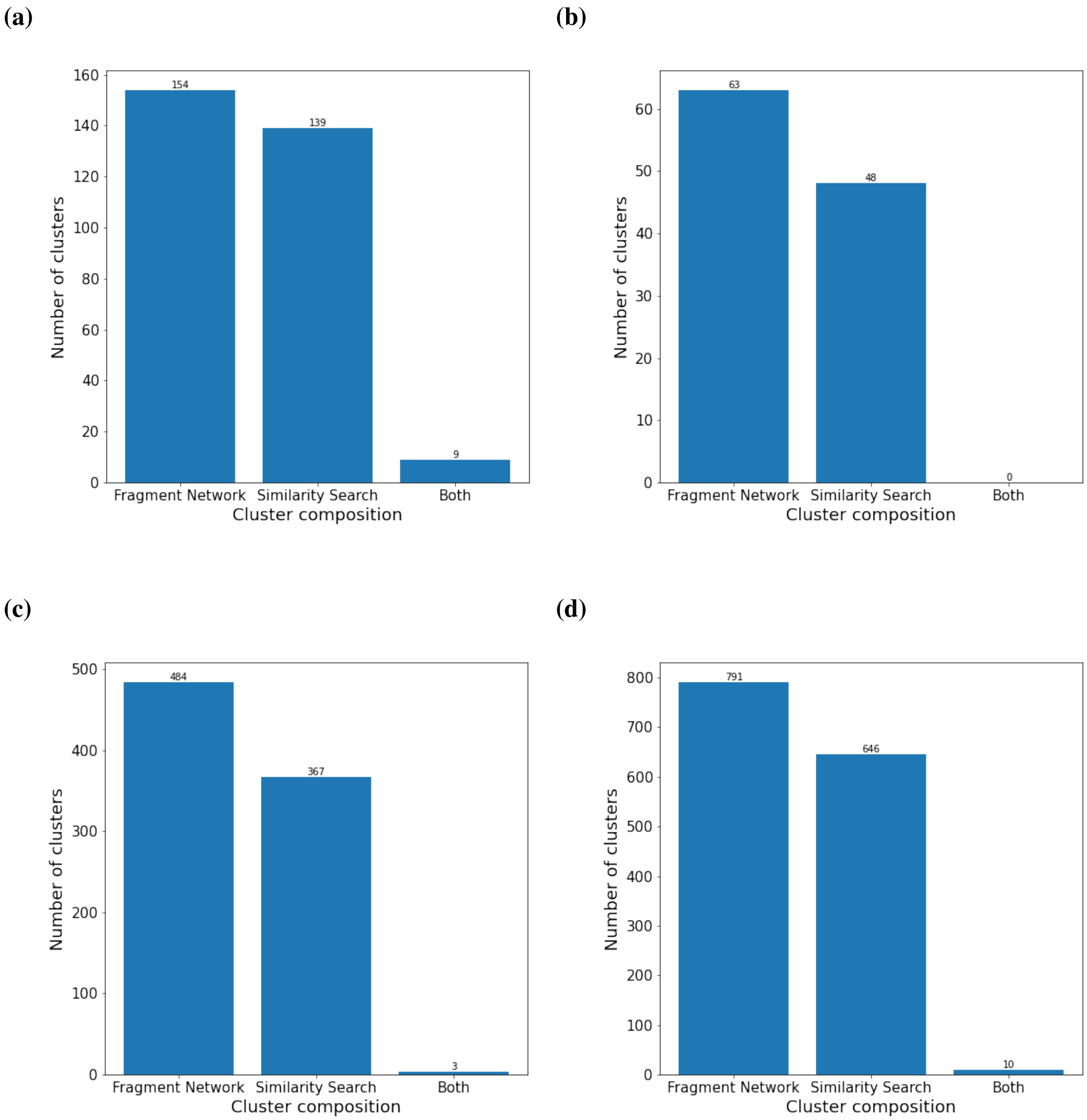

Figure S11: **Cluster composition after Butina clustering of filtered compounds.** The filtered compounds across targets (**a**) dipeptidyl peptidase 11 (DPP11), (**b**) poly(ADP-ribose) polymerase 14, (PARP14) (**c**) non-structural protein 13 (nsp13) and (**d**) main protease (Mpro) were clustered using Butina clustering (distance threshold of 0.3; calculated using Tanimoto and Morgan fingerprint with 2,048 bits and radius 2). The number of clusters containing only Fragment Network compounds, only similarity search compounds or both are shown.

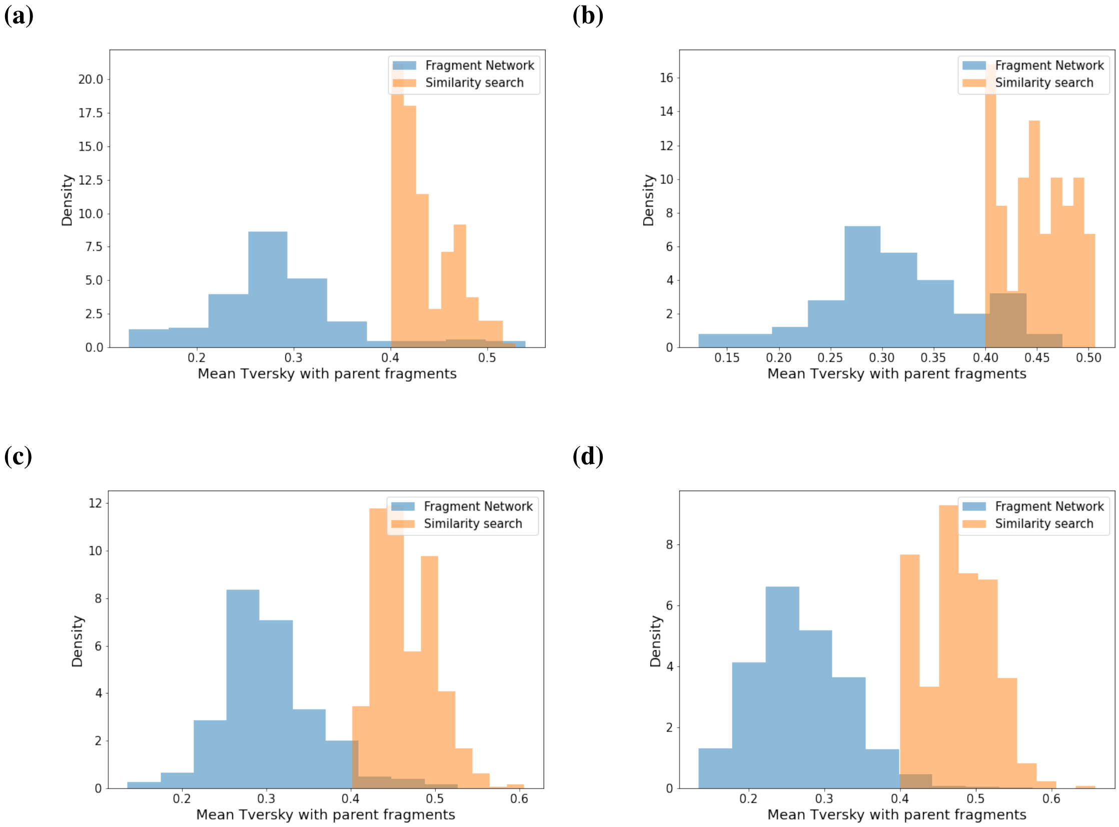

Figure S12: **The mean Tversky similarities between filtered merges and their parent fragments found using similarity search.** The mean Tversky similarity between similarity search-identified merges (after filtering) and their parent fragments are shown for targets (**a**) dipeptidyl peptidase 11 (DPP11), (**b**) poly(ADP-ribose) polymerase 14, (PARP14) (**c**) non-structural protein 13 (nsp13) and (**d**) main protease (Mpro).

(a) **(b)**

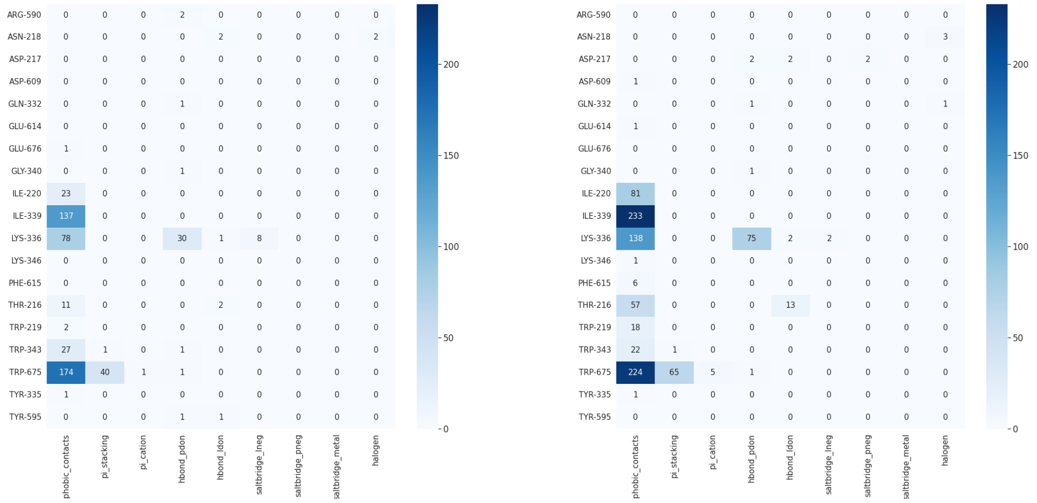

Figure S13: **Heatmaps showing the interactions made by filtered compounds from Fragment Network and similarity searches for DPP11.** All interactions were predicted using the Protein–Ligand Interaction Profiler (PLIP). The heatmaps show the counts for the numbers of each interaction type made in the filtered compound sets using the Fragment Network for dipeptidyl peptidase 11 (DPP11) using the (**a**) Fragment Network and (**b**) similarity searches.

(a) **b)**

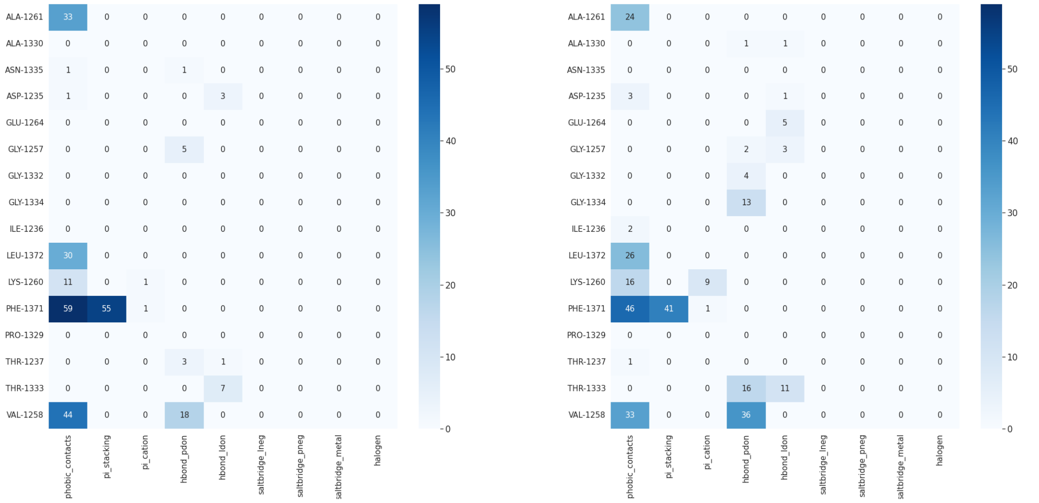

Figure S14: **Heatmaps showing the interactions made by filtered compounds from Fragment Network and similarity searches for PARP14.** All interactions were predicted using the Protein–Ligand Interaction Profiler (PLIP). The heatmaps show the counts for the numbers of each interaction type made in the filtered compound sets using the Fragment Network for poly(ADP-ribose) polymerase 14 (PARP14) using the (**a**) Fragment Network and (**b**) similarity searches.

(a) **(b)**

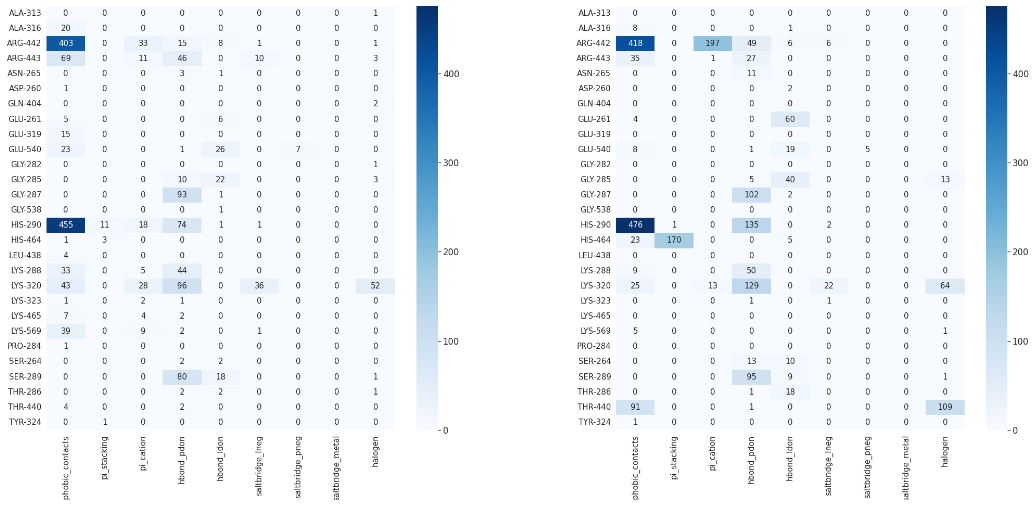

Figure S15: **Heatmaps showing the interactions made by filtered compounds from Fragment Network and similarity searches for nsp13.** All interactions were predicted using the Protein–Ligand Interaction Profiler (PLIP). The heatmaps show the counts for the numbers of each interaction type made in the filtered compound sets using the Fragment Network for non-structural protein 13 (nsp13) using the (**a**) Fragment Network and (**b**) similarity searches.

(a) **(b)**

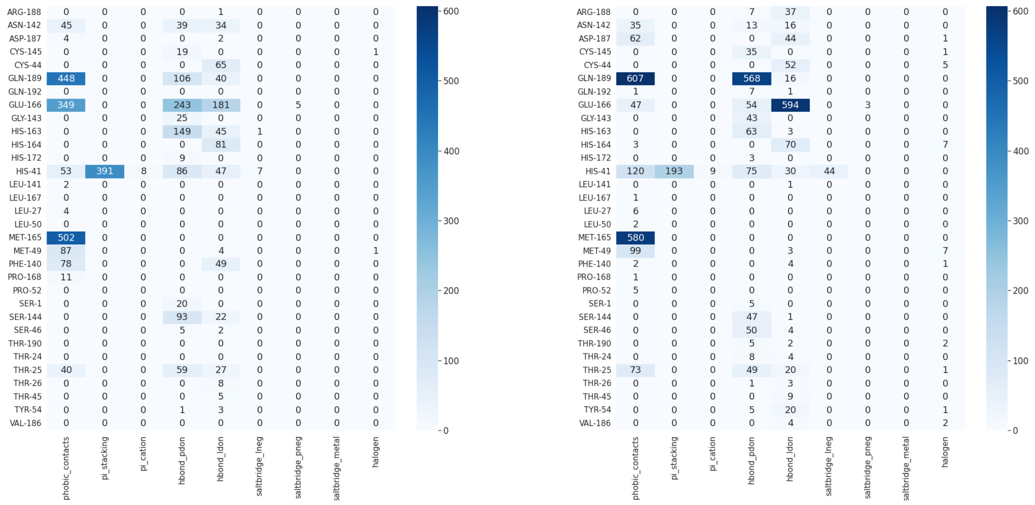

Figure S16: **Heatmaps showing the interactions made by filtered compounds from Fragment Network and similarity searches for Mpro.** All interactions were predicted using the Protein–Ligand Interaction Profiler (PLIP). The heatmaps show the counts for the numbers of each interaction type made in the filtered compound sets using the Fragment Network for main protease (Mpro) using the (**a**) Fragment Network and (**b**) similarity searches.

Table S6: The percentage of compounds removed by each filtering step.

Target	Filter	Fragment Network compounds: % compounds removed	Fragment Network compounds: % total compounds entering filter	Similarity search compounds: % compounds removed	Similarity search compounds: % total compounds entering filter
DPP11	Descriptor	0.0	0.0	0.0	0.0
	Non-ring bond	17.6	17.6	3.1	3.1
	Expansion	1.0	1.2	–	–
	Embedding	60.8	74.7	79.6	82.1
	Overlap	3.6	17.7	2.0	11.7
	Fragmenstein	16.4	96.8	15.0	98.2
	Energy of pose	0.1	9.7	0.0	7.2
PARP14	Descriptor	0.1	0.1	0.1	0.1
	Non-ring bond	28.0	28.1	19.2	19.2
	Expansion	0.1	0.2	–	–
	Embedding	51.5	71.8	75.0	92.9
	Overlap	8.9	43.9	1.8	32.2
	Fragmenstein	11.2	98.9	3.8	98.7
	Energy of pose	0.1	50.7	0.0	24.3
nsp13	Descriptor	3.9	3.9	0.2	0.2
	Non-ring bond	51.0	53.0	24.0	24.1
	Expansion	0.0	0.0	–	–
	Embedding	21.3	47.2	62.0	81.8
	Overlap	7.6	31.8	3.5	25.1
	Fragmenstein	15.2	93.9	9.4	90.8
	Energy of pose	0.1	9.3	0.1	7.1
Mpro	Descriptor	0.8	0.8	0.0	0.0
	Non-ring bond	27.6	27.9	11.9	11.9
	Expansion	0.3	0.5	–	–
	Embedding	52.1	73.1	74.5	84.6
	Overlap	6.2	32.7	4.9	36.3
	Fragmenstein	12.1	93.8	8.1	93.3
	Energy of pose	0.2	28.7	0.2	33.9

Table S7: CPU time required to run entire filtering pipeline (and Fragmenstein time alone).

Target	Technique	Total filtering time (CPU days)	Total Fragmenstein time (CPU days)	Number of mols filtered	Number of mols through Fragmenstein
DPP11	Fragment Network	23.1	19.6	40,553	6,968
	Similarity search	53.0	46.0	116,819	16,451
PARP14	Fragment Network	13.0	11.6	116,084	13,336
	Similarity search	6.3	5.2	175,376	5,792
nsp13	Fragment Network	19.2	16.4	53,618	8,616
	Similarity search	16.4	13.0	88,539	7,203
Mpro	Fragment Network	75.9	64.7	175,024	23,093
	Similarity search	55.4	43.3	261,756	20,165

Table S8: Top-scoring compounds for fragment pairs where both techniques identify filtered compounds.

Target	Number of FN top compounds	Number of SS top compounds	Total pairs
DPP11	3	9	12
PARP14	3	3	6
nsp13	2	4	6
Mpro	17	19	36

smina was used to calculate the docking scores for the poses of the filtered compounds generated with Fragmenstein. The default scoring function is used and the minimized affinity for each filtered compound was recorded. Docking scores were also normalized according to heavy atom count to reflect ligand efficiency values. Owing to the lack of fragment pairs for which both search techniques produce comparable numbers of filtered compounds, the table shows, for pairs where both techniques identify filtered compound(s), which technique resulted in the top-scoring compound.

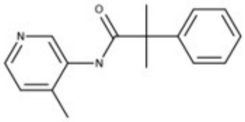

Tanimoto: 0.73

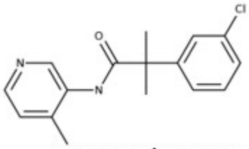

JAN-GHE-83b26c96-22

F-IC50: 96.9μM
RF-IC50: 24.5μM

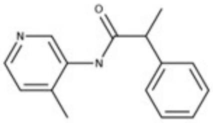

Tanimoto: 0.73

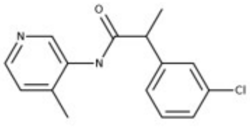

TRY-UNI-714a760b-18

F-IC50: 26.2μM
RF-IC50: 13.0μM

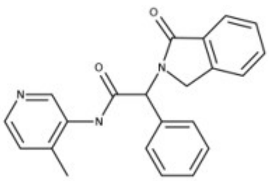

Tanimoto: 0.70

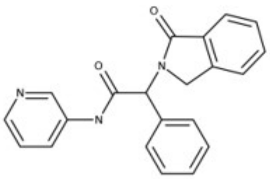

LON-WEI-b2874fec-25

F-IC50: 99.5μM
RF-IC50: 59.6.0μM

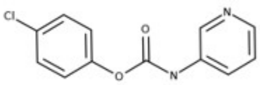

Tanimoto: 0.68

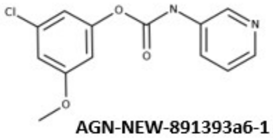

AGN-NEW-891393a6-1

F-IC$_{50}$: 6.7μM
RF-IC50: 1.5μM

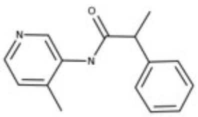

Tanimoto: 0.68

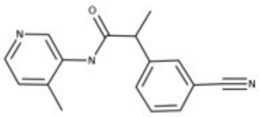

TRY-UNI-714a760b-22

F-IC$_{50}$: 54.9μM
RF-IC50: 45.3μM

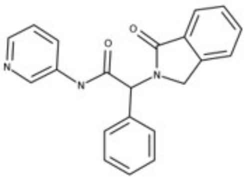

Tanimoto: 0.68

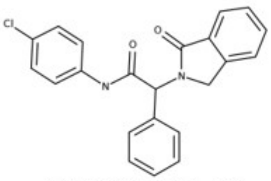

LON-WEI-b2874fec-16

F-IC$_{50}$: 99.5μM
RF-IC50: 65.0μM

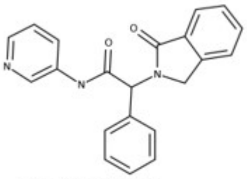

Tanimoto: 0.67

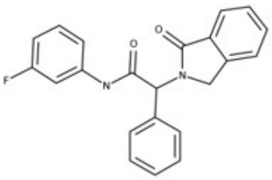

LON-WEI-b2874fec-24

F-IC$_{50}$: 99.5μM
RF-IC50: 59.9μM

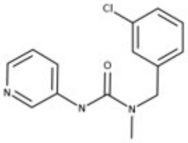

Tanimoto: 0.65

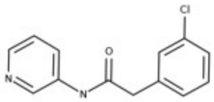

EDG-MED-0da5ad92-2

F-IC$_{50}$: 53.7μM
RF-IC50: 99.0μM

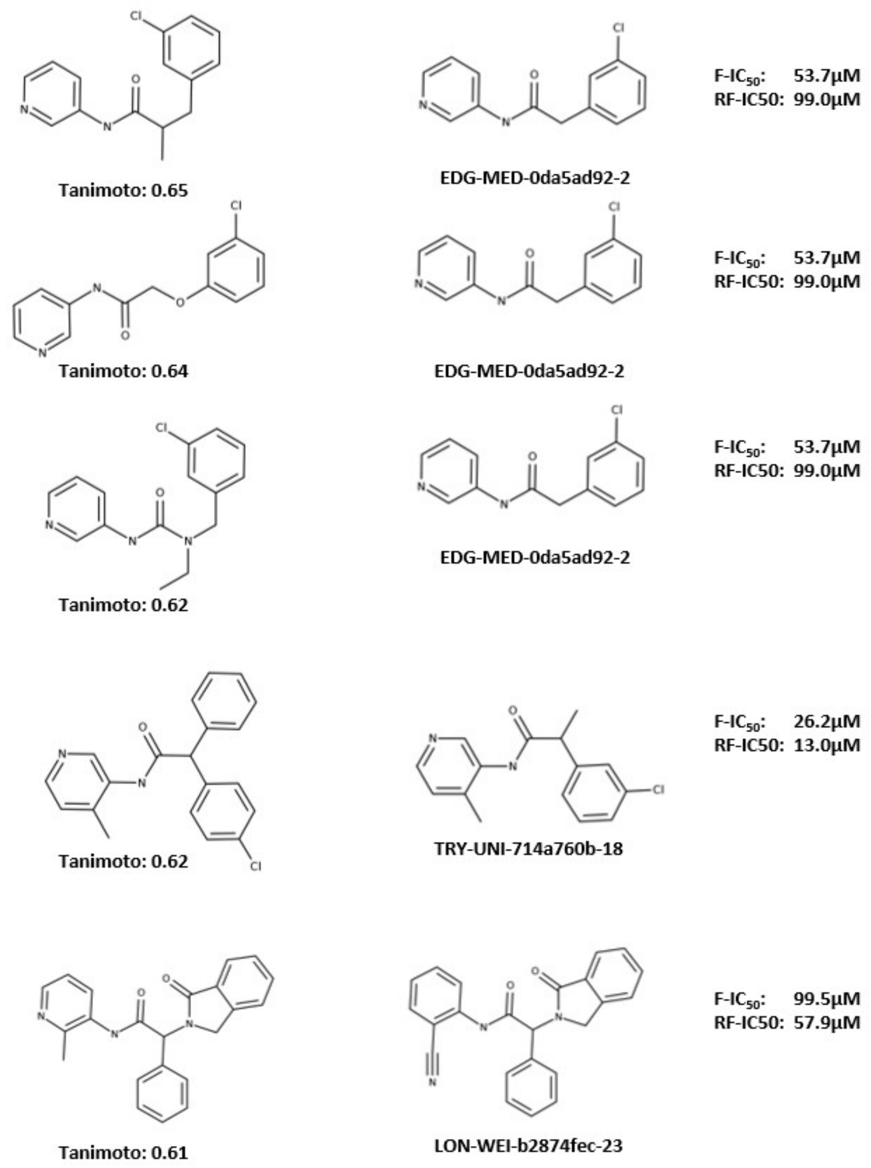

Figure S17: **The Fragment Network identifies close analogues to known Mpro inhibitors.** Example compounds identified using the Fragment Network (left-hand side) and similar compounds with IC_{50} values within the micromolar range, recorded using either a fluorescence assay (F-IC_{50}) or RapidFire mass spectrometry (RFIC_{50}). Similar compounds were identified by calculating the Tanimoto similarity using Morgan fingerprints (radius2; 2,048 bits).

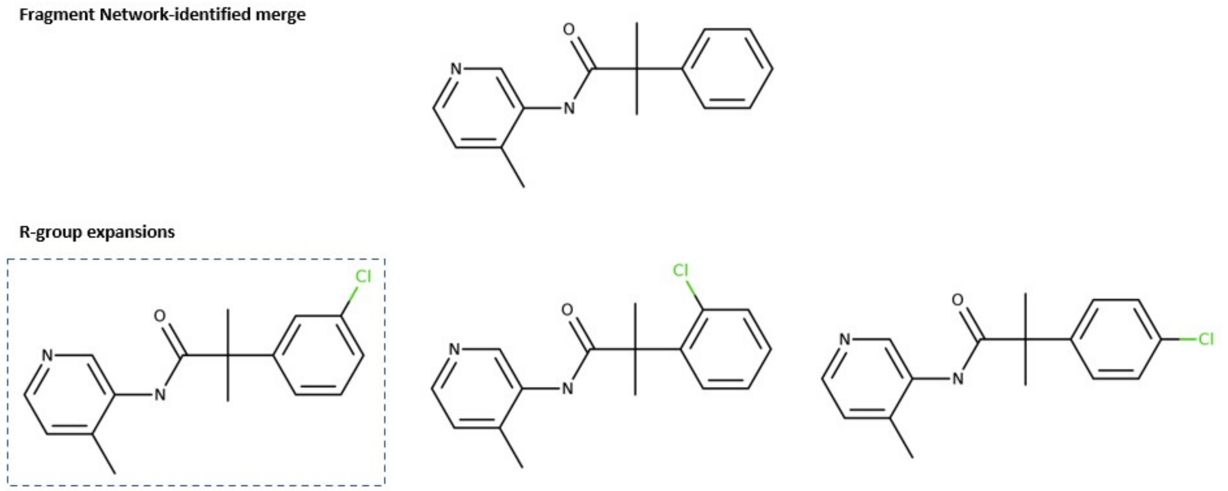

Figure S18: **R-group expansion retrieves inhibitor JAN-GHE-83b26c96-22.** R-group expansion of a Fragment Network-identified merge identifies a known inhibitor with a RapidFire mass spectrometry IC_{50} value of 24.5μM.

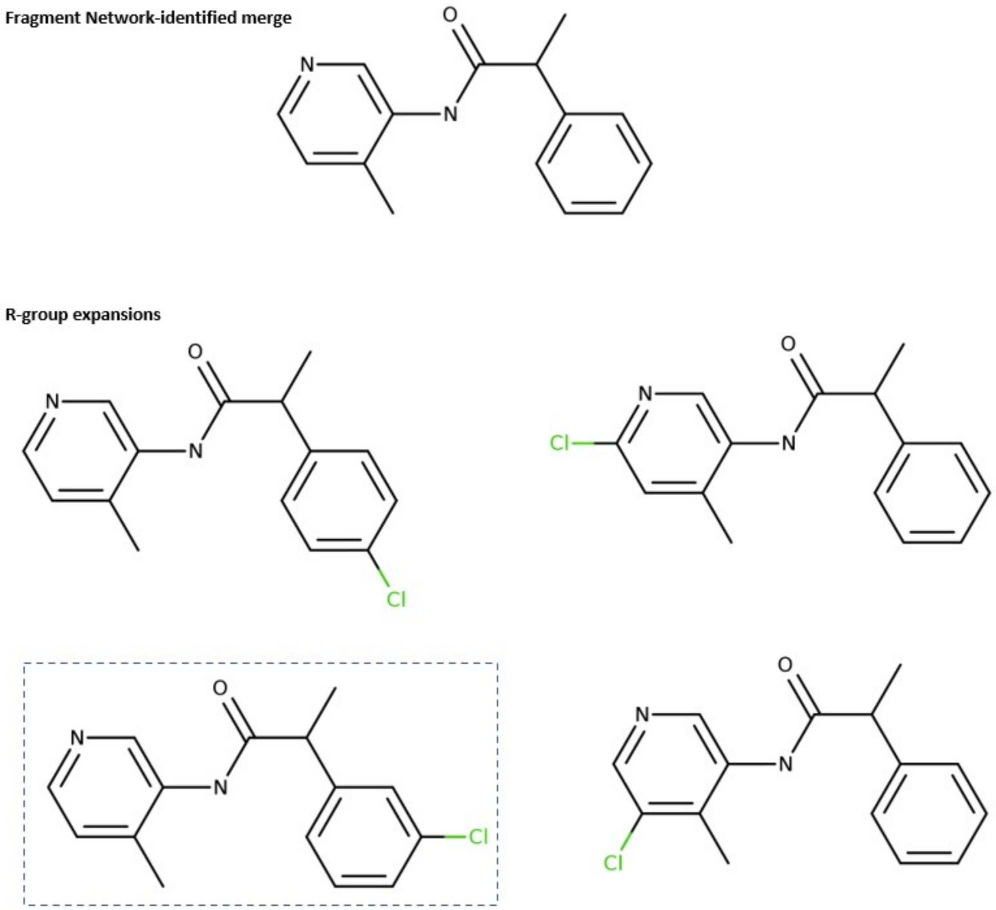

Figure S19: **R-group expansion retrieves inhibitor TRY-UNI-714a760b-18.** R-group expansion of a Fragment Network-identified merge identifies a known inhibitor with a fluorescence assay IC_{50} value of 25.2μM and a RapidFire mass spectrometry IC_{50} value of 13.0μM.

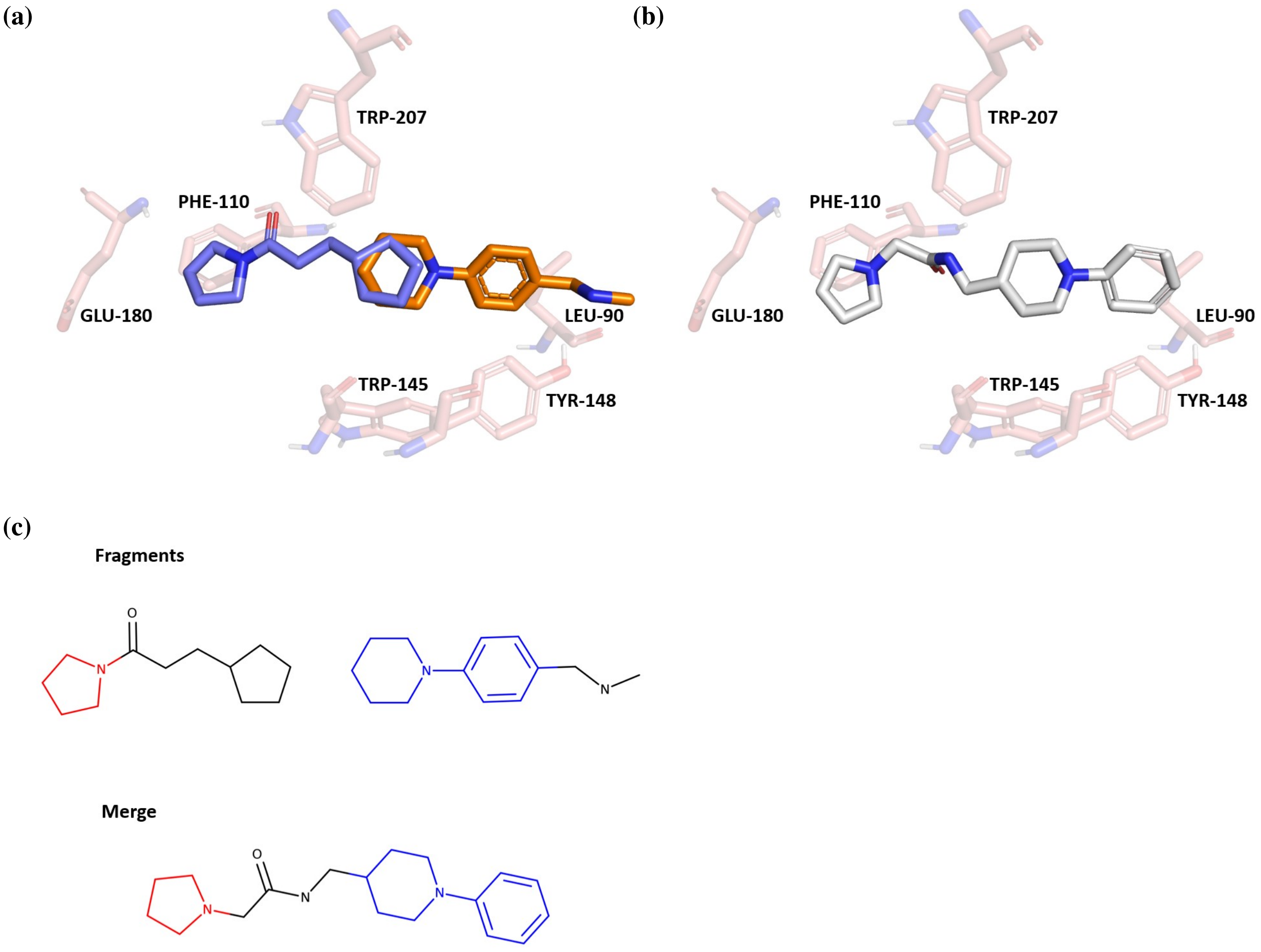

Figure S20: **An example merge against EthR found using the entire filtering pipeline.** (**a**) The crystal structures of two parent fragments against EthR. (**b**) The orientation of the merge (white) generated using Fragmenstein. (**c**) The fragments and merge in 2D; colours indicate the substructures used in forming the merge.

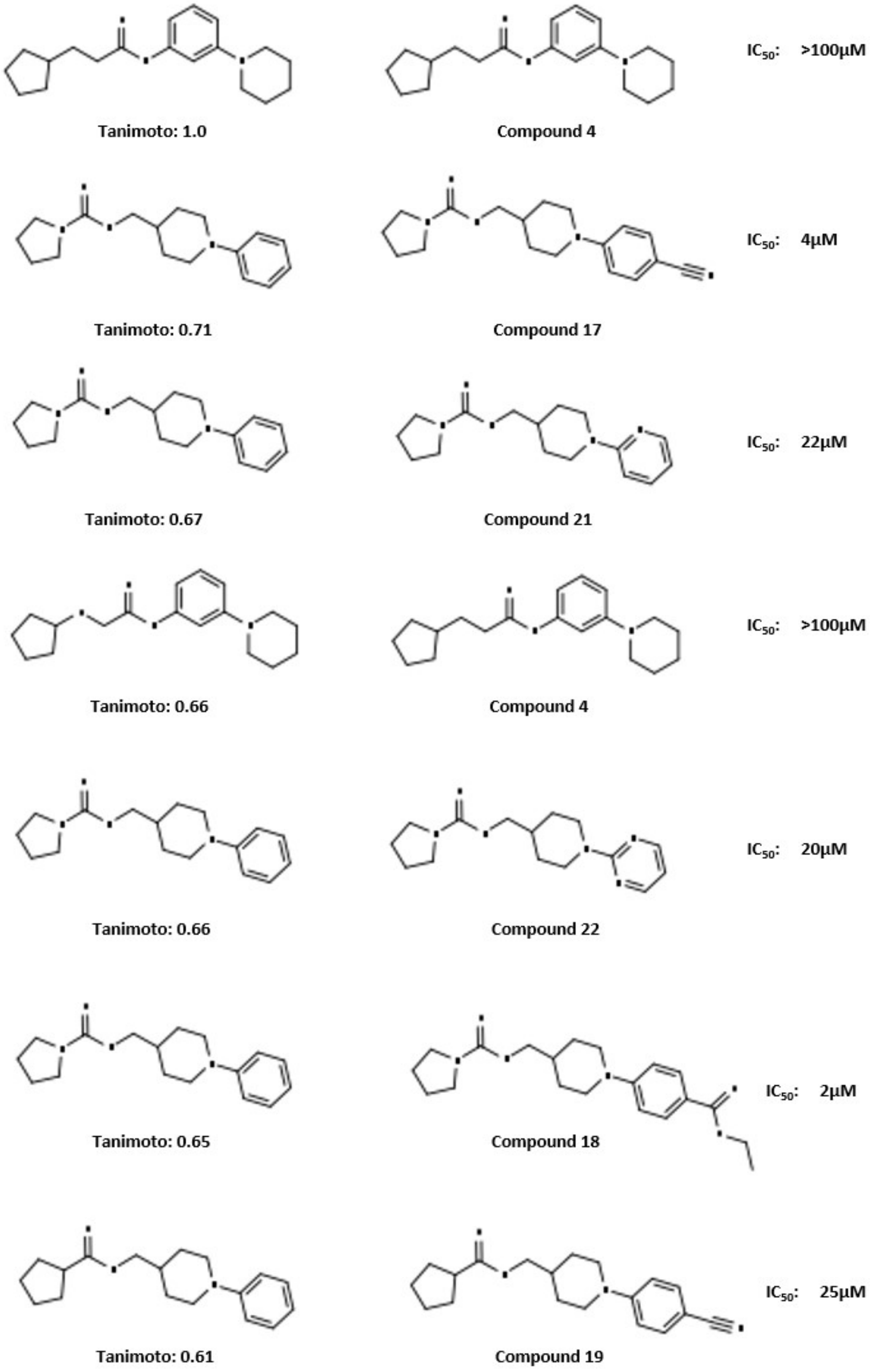

Figure S21: **The Fragment Network identifies close analogues to known EthR inhibitors.** Example compounds identified using the Fragment Network (left-hand side) and similar compounds with IC_{50} values recorded using surface plasmon resonance. Similar compounds were identified by calculating the Tanimoto similarity using Morgan fingerprints (radius2; 2,048 bits).
